# Transcriptomic analysis links diverse hypothalamic cell types to fibroblast growth factor 1-induced sustained diabetes remission

**DOI:** 10.1038/s41467-020-17720-5

**Published:** 2020-09-07

**Authors:** Marie A. Bentsen, Dylan M. Rausch, Zaman Mirzadeh, Kenjiro Muta, Jarrad M. Scarlett, Jenny M. Brown, Vicente Herranz-Pérez, Arian F. Baquero, Jonatan Thompson, Kimberly M. Alonge, Chelsea L. Faber, Karl J. Kaiyala, Camdin Bennett, Charles Pyke, Cecilia Ratner, Kristoffer L. Egerod, Birgitte Holst, Thomas H. Meek, Burak Kutlu, Yu Zhang, Thomas Sparso, Kevin L. Grove, Gregory J. Morton, Birgitte R. Kornum, José-Manuel García-Verdugo, Anna Secher, Rasmus Jorgensen, Michael W. Schwartz, Tune H. Pers

**Affiliations:** 1 UW Medicine Diabetes Institute, University of Washington, Seattle, WA USA; 2 Novo Nordisk Foundation Center for Basic Metabolic Research, Faculty of Health and Medical Sciences, University of Copenhagen, Copenhagen, Denmark; 3 Barrow Neurological Institute, Phoenix, AZ USA; 4 Department of Pediatric Gastroenterology and Hepatology, Seattle Children’s Hospital, Seattle, WA USA; 5 Cavanilles Institute of Biodiversity and Evolutionary Biology, University of Valencia, Valencia, Spain; 6 Predepartamental Unit of Medicine, Jaume I University, Castelló de la Plana, Spain; 7 Obesity Research Unit, Novo Nordisk Research Center Seattle, Inc., Seattle, WA USA; 8 Department of Oral Health Sciences, School of Dentistry, University of Washington, Seattle, WA USA; 9 Pathology & Imaging, Global Discovery and Development Sciences, Novo Nordisk A/S, Maaloev, Denmark; 10 Bioinformatics and Data Mining, Global Research Technologies, Novo Nordisk A/S, Maaloev, Denmark; 11 Department of Neuroscience, University of Copenhagen, Copenhagen, Denmark; 12 Diabetes Research, Global Drug Discovery, Novo Nordisk A/S, Maaloev, Denmark; 13 Cytoki Pharma, Copenhagen, Denmark; 14 Present Address: Chakri Naruebodindra Medical Institute, Faculty of Medicine, Ramathibodi Hospital, Mahidol University, Bangkok, Thailand

**Keywords:** Gene expression analysis, Computational models, Neuroscience, Type 2 diabetes

## Abstract

In rodent models of type 2 diabetes (T2D), sustained remission of hyperglycemia can be induced by a single intracerebroventricular (icv) injection of fibroblast growth factor 1 (FGF1), and the mediobasal hypothalamus (MBH) was recently implicated as the brain area responsible for this effect. To better understand the cellular response to FGF1 in the MBH, we sequenced >79,000 single-cell transcriptomes from the hypothalamus of diabetic Lep^*ob/ob*^ mice obtained on Days 1 and 5 after icv injection of either FGF1 or vehicle. A wide range of transcriptional responses to FGF1 was observed across diverse hypothalamic cell types, with glial cell types responding much more robustly than neurons at both time points. Tanycytes and ependymal cells were the most FGF1-responsive cell type at Day 1, but astrocytes and oligodendrocyte lineage cells subsequently became more responsive. Based on histochemical and ultrastructural evidence of enhanced cell-cell interactions between astrocytes and Agrp neurons (key components of the melanocortin system), we performed a series of studies showing that intact melanocortin signaling is required for the sustained antidiabetic action of FGF1. These data collectively suggest that hypothalamic glial cells are leading targets for the effects of FGF1 and that sustained diabetes remission is dependent on intact melanocortin signaling.

## Abstract (web summary)

In rodent models of type 2 diabetes, sustained remission of hyperglycemia can be induced by FGF1 action in the mediobasal hypothalamus. Here, the authors show that FGF1-injection is followed by marked changes in glial cell populations and that the sustained glycemic response is dependent on intact melanocortin signaling.

## Introduction

Type 2 diabetes (T2D) is among the most common and costly biomedical challenges confronting modern society^1^. Standard treatment of this disease involves daily dosing of one or more antidiabetic drugs and can be effective but fails to deliver adequate glycemic control to a majority of patients^2^. A compelling need for more effective treatment modalities therefore exists, and the brain has emerged as an important target for research in this area^3^.

We recently reported that, in Lep^*ob/ob*^ mice and other rodent models of T2D, sustained diabetes remission can be achieved by a single intracerebroventricular (icv) injection of fibroblast growth factor 1 (FGF1)^4^, and the hypothalamus has been identified as the brain area responsible for this FGF1 effect^5^. While this finding is in line with accumulating evidence supporting a key role for the hypothalamus in glucose homeostasis, our current understanding of glucoregulatory neurocircuitry is limited^6,7^. Most of this work is focused on hypothalamic neurons with glucose-sensing properties^8^, but many glial cell types are also potential targets for the hypothalamic action of FGF1, since tanycytes and astrocytes are implicated in glucose homeostasis^9,10^ and since glial cell responsiveness to FGF ligands is well documented^5,11,12^. Moreover, glia–neuron interactions can modify neuronal activity and remodel neurocircuit structure^13–15^, effects of interest for understanding the long-lived nature of the FGF1 effect.

As an unbiased approach to measuring gene expression within single cells in the hypothalamus^16–18^, single-cell RNA sequencing (scRNA-seq) provides a useful strategy both for identifying FGF1-responsive cell types in this brain area and for characterizing their response to icv FGF1 administration at the transcriptional level. With this goal in mind, we performed this analysis across several time points following a single icv injection of FGF1 or vehicle in diabetic Lep^*ob/ob*^ mice. Using a combination of scRNA-seq and histological techniques, we report that glial responses to FGF1 were generally rapid in onset and accompanied by an activated morphology. We also identified Agrp neurons, key components of the melanocortin signaling pathway, as the most FGF1-responsive neuronal cell type at the transcriptional level, and in a series of in vivo studies, we show that sustained diabetes remission induced by icv FGF1 is dependent on intact melanocortin receptor signaling.

## Results

### Study design

**Fig. 1:**
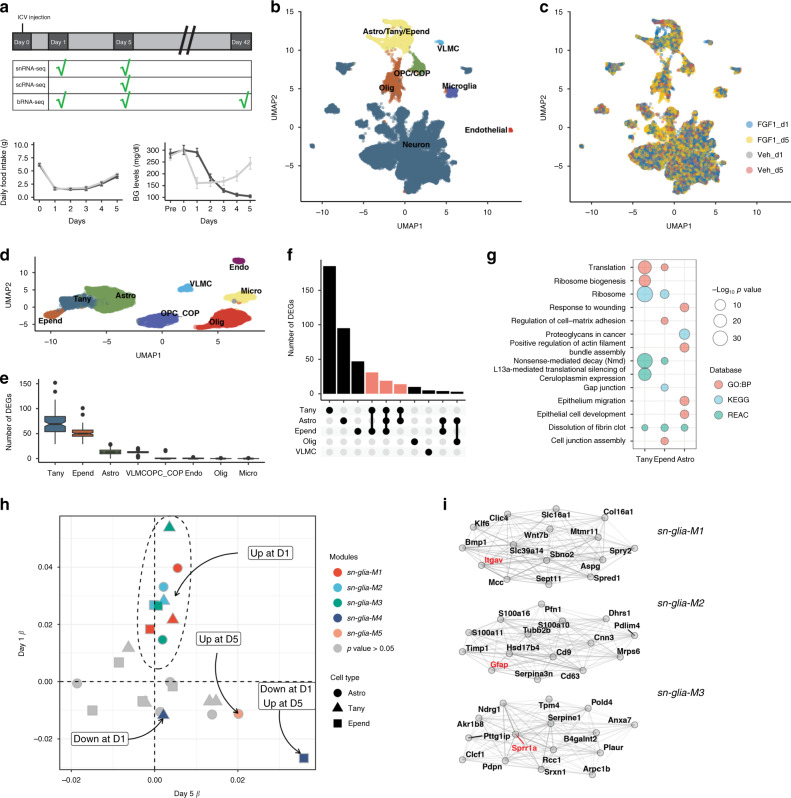
Study design and icv FGF1-induced temporal glia responses. **a** Diabetic Lep^*ob/ob*^ mice received a single intracerebroventricular (icv) injection of FGF1 (3 µg) or vehicle at Day 0. Hypothalami were harvested on Days 1, 5, and 42 after icv injection. Vehicle-injected mice were pair-fed (PF) to the amount of food consumed by icv FGF1-injected Lep^*ob/ob*^ mice and morning blood glucose levels were monitored. Plots are represented as mean ± SEM, and source data are provided as a Source data file; FGF1 (*n* = 17), vehicle (*n* = 15). **b** Uniform manifold approximation and projection (UMAP) clustering of hypothalamic nuclei from Day 1 and Day 5 snRNA-seq datasets. Source data are available through the NCBI Gene Expression Omnibus (GEO; GSE153551). **c** UMAP clustering from **b** colored according to treatment group and time of euthanasia. **d** UMAP clustering of hypothalamic non-neuronal cells from Day 1 and Day 5 snRNA-seq datasets. **e** Number of differentially expressed genes (DEGs) for non-neuronal subclusters at Day 1 identified through repeated downsampling (10 cells/group/cell type/iteration; 100 iterations), *n* = 3 mice/group. Data are presented as median with interquartile range (IQR) and summary statistics are provided as a Source data file. **f** Each bar represents the number of unique DEGs for one or a certain combination of cell types (pseudobulk approach, see “Methods”) from Day 1 snRNA-seq dataset, *n* = 3 mice/group. **g** Associated Gene Ontology terms (biological process category; red), KEGG (blue) and REACTOME (green) pathways associated with DEGs in the indicated cell types. Point size indicates −log_10_(*p* value) (Bonferroni adjusted). **h** Weighted gene co-expression network analysis (WGCNA) of tanycytes, ependymal cells, and astrocytes from Days 1 and 5 snRNA-seq datasets. Significantly changed gene modules (linear mixed-effects model; FDR < 0.05) are indicated with color and cell-specific expression is indicated by shape. Modules are plotted according to their *β*-values; Day 1 (*n* = 3/group), Day 5 (*n* = 2/group). **i** Module plots of top 15 genes of *sn-glia-M1*–*sn-glia-M3*, which are upregulated on Day 1. Genes in red denote genes mentioned in the text. Astro astrocytes, Tany tanycytes, Epend ependymal cells, Olig oligodendrocytes, OPC oligodendrocyte precursor cell, COP committed to differentiate oligodendrocyte, VLMC vascular leptomeningeal cell.

Consistent with previous studies^4,5,11^, icv delivery of FGF1 (3 µg) into the lateral ventricle of diabetic Lep^*ob/ob*^ mice induced a robust but transient anorectic response at Day 1, with normalization of blood glucose (BG) levels occurring by Day 5 (Fig. 1a). To identify transcriptional correlates of these distinct responses to FGF1, we generated a series of RNA-seq datasets at Days 1, 5, and 42 (Fig. 1a) and adjusted for differences of food intake by pair-feeding of icv vehicle-injected controls to the intake of icv FGF1-injected mice.

To transcriptionally characterize Days 1 and 5, we performed single-nucleus RNA-sequencing (snRNA-seq) and generated a dataset comprising 15,291 cells that clustered into 9 overall cell populations (Fig. 1b, c). For this dataset, we, on average, recovered 3258 transcripts (unique molecular identifiers (UMIs)) and 1,792 unique genes per cell across all hypothalamic cell populations.

### Tanycytes, ependymal, and astrocytes are highly responsive to icv FGF1

To better characterize the response of hypothalamic tanycytes, astrocytes, and other glial cells Day 1 after icv FGF1 injection in diabetic Lep^*ob/ob*^ mice, we extracted all non-neuronal cells and re-clustered them to delineate glial subpopulations (Fig. 1d). To estimate which subpopulations were most affected one day after icv FGF1 injection, differentially expressed genes (DEGs) were identified with a repeated downsampling approach to control for both cell number and between-sample variation^19^. In line with previous evidence that both tanycytes and astrocytes are highly responsive to FGF1^4,5,11^, robust transcriptomic responses were observed in tanycytes, ependymal cells, and, to a lesser degree, astrocytes (false discovery rate (FDR) < 0.1; Fig. 1e). Using a pseudobulk approach to identify DEGs (see “Methods”), our analysis of FGF1-induced transcriptional changes identified a core set of genes (*n* = 21) shared between tanycytes, ependymal cells, and astrocytes, suggesting a common response across these cell types (Fig. 1f, Supplementary Data 1). Furthermore, we tested for enrichment of genesets in the Gene Ontology biological process (GO:BP)^20^, REACTOME (REAC)^21^, and Kyoto Encyclopedia of Genes and Genomes (KEGG)^22^ databases (Fig. 1g; Supplementary Data 2). Tanycytes and ependymal cells showed strong upregulation of ribosomal pathways and downregulation of cilia activity, whereas astrocytes showed increased activity in processes involved in tissue remodeling compared to pair-fed, vehicle-treated mice.

To further characterize both the biological pathways induced by FGF1 across tanycytes, ependymal cells, and astrocytes and how these responses change over time, we performed weighted gene co-expression network analysis (WGCNA)^23^ on these glial subsets. Of the nine modules identified, three were significantly increased by icv FGF1 at Day 1 (Fig. 1h). Modules *sn-glia-M1* and *sn-glia-M2* were increased by FGF1 to a similar extent across all three cell types, whereas *sn-glia-M3* appeared to be more strongly upregulated in tanycytes than the other cell types (Fig. 1h; linear mixed-effects models; FDR < 0.05; Supplementary Data 3). Consistent with the ability of FGF1 to signal through the extracellular signal-related kinase 1/2 (ERK1/2) pathway^5^, the *sn-glia-M1* module was significantly enriched for “mitogen-activated protein kinase (MAPK) cascade” and “FGF receptor 1 signaling” pathways and included the gene *Itgav* encoding an integrin subunit shown to modulate FGF signaling^24^. Furthermore, module *sn-glia-M2*, which included *Gfap* and *Vim* (markers of reactive astrocytes^25^), was upregulated across all three cell populations (Fig. 1h), suggesting a pan-reactive glial response. Module *sn-glia-M3* was strongly induced in tanycytes and contains the gene *Sprr1a* (Fig. 1i), which marks a unique subset of tanycytes situated at the junction of the arcuate nucleus (ARC) and the median eminence (ME)^16^.

Whereas these three modules were strongly induced Day 1 after icv FGF1 injection, each of these responses was nearly normalized by Day 5, with a different set of modules showing increased expression relative to vehicle at this time point. Among the modules upregulated Day 5 were *sn-glia-M4* and *sn-glia-M5*, which were activated specifically in astrocytes and ependymal cells, respectively (Fig. 1h). These data suggest that the transcriptomic response to FGF1 evolves over time in a manner that differs fundamentally between astrocyte, tanycyte, and ependymal cell populations.

### Tanycyte activation is sustained through Day 5 following icv FGF1 injection

**Fig. 2:**
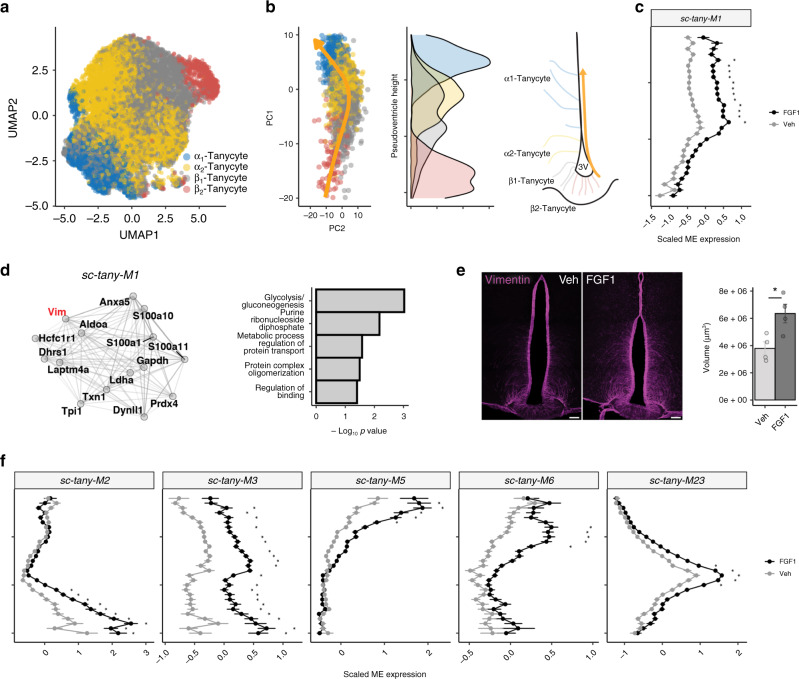
Tanycyte module expression maps to the spatial distribution lining the third ventricle. **a** UMAP clustering of tanycytes (β2-, β1-, α1-, α2-Tanycytes) from the Day 5 single-cell RNA-sequencing (scRNA-seq) dataset. Cell type annotations were inferred from a published dataset^16^. **b** Tanycyte lineage cells plotted on principal components 1 and 2 revealed a trajectory (left). The colors represent the subtype of each cell from **a**. A principal curve fit across the trajectory was used to assign a pseudoventricle score to each cell corresponding to the spatial location of that cell type along third ventricular (3V) wall. Density plot of each tanycyte subpopulation plotted along the pseudoventricle revealed a continuum of gene expression and non-distinct shifts in the expression of marker genes along the pseudoventricle. **c** Scaled module expression of *sc-tany-M1* in FGF1-treated (black) and vehicle (gray) tanycytes (linear mixed-effects model; bin size of 1; mean ± SEM; *p < 0.05) (Bonferroni adjusted) from the Day 5 scRNA-seq dataset (*n* = 6 mice/group). Exact adjusted *p* values are provided in Supplementary Data 5. ME module eigengene. **d** Gene–gene network plot of the correlation of top 15 genes in module *sc-tany-M1* (left) and associated pathways (right). Genes mentioned in the text are labeled in red. **e** Representative images of immunolabeling of vimentin in diabetic Lep^*ob/ob*^ mice 1 week after vehicle or icv FGF1 injection. Pixel dimensions: 0.27 × 0.27 × 2.00 microns. Quantification: dots represent volume of vimentin-positive voxels assessed by iMARIS for individual animals (mean from 2 sections per animal, *n* = 4/group), mean ± SEM. Unpaired *t* test, two tailed, *p = 0.022 (not adjusted). Source data are provided as a Source data file. **f** Scaled module expression of *sc-tany-M2*, *sc-tany-M3*, *sc-tany-M5*, *sc-tany-M6*, and *sc-tany-M23* with 5 consecutive points of difference in expression along the pseudoventricle between FGF1-treated (black) and vehicle (gray) tanycytes (linear mixed-effects models; bin size of 1; mean ± SEM; *p < 0.05) (Bonferroni adjusted) from the Day 5 scRNA-seq dataset (*n* = 6 mice/group). Exact adjusted *p* values are provided in Supplementary Data 5.

As tanycyte and ependymal cell responses diverged with the passage of time following icv FGF1 injection, we generated a larger single-cell RNA-sequencing (scRNA-seq) Day 5 dataset, which comprised 63,904 hypothalamic cells from a separate cohort of icv FGF1-injected Lep^*ob/ob*^ mice and PF, icv vehicle-injected controls (*n* = 6/group). After quality control and doublet removal, cells were clustered into 14 cell populations (Supplementary Fig. 1). On average, we recovered 1802 UMIs and 966 unique genes/cell across all cell populations. Within this larger dataset, we first inspected cell types lining the third ventricle (3V) (Supplementary Fig. 2). Subclustering identified the four major tanycyte subpopulations: α1-, α2-, β1-, and β2-tanycytes (Fig. 2a). Because tanycyte cell identity and function vary with dorsal–ventral distribution along the ventricular wall, we anticipated finding a continuum of gene expression moving dorsally up the 3V. We therefore plotted all tanycyte cells in principal component (PC) space, which revealed a one-dimensional curve, reminiscent of what is seen in terminally differentiated cells with spatial zonation^26^. We next fit a principal curve to our data and assigned each cell a value on that curve, henceforth referred to as “pseudoventricle score” (Fig. 2b). Application of WGCNA across this tanycyte population identified 35 modules (Supplementary Data 5), of which 7 showed a significant difference following icv FGF1 treatment compared to vehicle across at least 5 consecutive points along the pseudoventricle. Module *sc-tany-M1* showed a strong significant (linear mixed-effects model; FDR < 0.05) increase in expression excluding locations in which β2/β1-tanycyte marker expression was high. This module contained the gene *Vim* and enriched for pathways such as “glycolysis” and “gluconeogenesis” (Fig. 2c, d), suggesting a shift in metabolic processes in these cells. Immunostaining for vimentin confirmed a robust increase in both ependymal and tanycyte vimentin content 1 week after icv FGF1 injection at the predicted ventricle heights. Combined with a marked increase in the number of tanycyte processes projecting into adjacent hypothalamic parenchyma, these findings are highly suggestive of a phenotype switch in these cells (Fig. 2e). Furthermore, we found that *sc-tany-M2*, which contained genes with stem cell-like properties^27,28^, was increased specifically in the ventral part of the pseudoventricle, and *sc-tany-M3* exhibited a significant FGF1-induced increase in expression across nearly the entire pseudoventricle and comprised genes involved in protein synthesis thus mirroring the response of tanycytes on Day 1. Modules *sc-tany-M5* and *sc-tany-M6* were expressed dorsally along the pseudoventricle, included *Cd24a* and *Igfbp5*, respectively, confirmed by in situ hybridization in cells lining the 3V (Supplementary Fig. 3)^29^, and identified in progenitor cells in the ventricular–subventricular zone^30^. Genes of the peroxiredoxin family characteristic of *sc-tany-*M23 exhibited a distinct expression pattern characteristic of α2-tanycytes, suggesting a role for this tanycyte subset in the hypothalamic response to oxidative stress that is upregulated by FGF1 (Fig. 2f). Additional studies are warranted to determine the contribution made by various tanycyte subsets to FGF1-induced diabetes remission (for more detailed results and a full list of tanycyte and ependymal modules, please refer to Supplementary Notes, Supplementary Fig. 4, and Supplementary Data 4 and 5).

### Evolution of the astrocyte response to icv FGF1

**Fig. 3:**
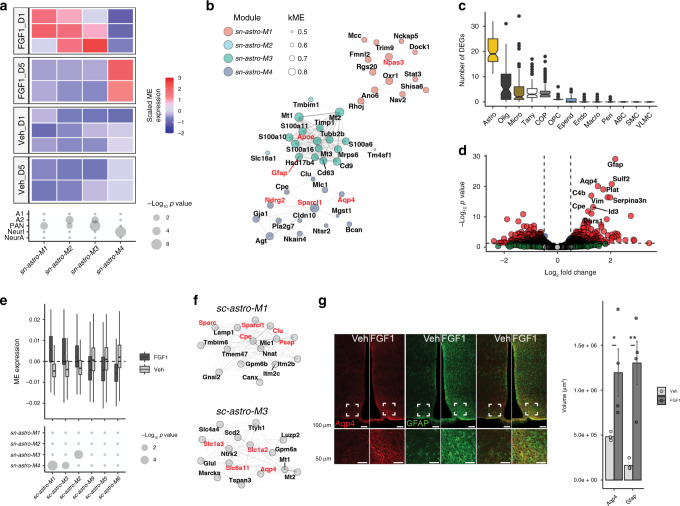
Evolution of the astrocyte response to icv FGF1. **a** Identification of *sn-astro-M1*–*sn-astro-M3* upregulated at Day 1 and *sn-astro-M4* upregulated at Day 5 in FGF1-treated mice (linear mixed-effects model, FDR < 0.05) using WGCNA on astrocytes from the snRNA-seq datasets. Enrichment of DEGs in astrocytes in response to lipopolysaccharide (neurotoxic, A1), middle cerebral artery occlusion (neuroprotective, A2), or overlapping genes (PAN)^25^ or enrichment of genes induced by neuron interaction (NeurI) or neuron activity (NeurA)^31^. Point size is scaled by −log_10_(*p* value) (Bonferroni adjusted). **b** Correlation of top 15 genes of *sn-astro-M1*–*sn-astro-M4*. Node size; correlation of the given gene with the module eigengene (kME). **c** Number of DEGs for non-neuronal subclusters identified in the Day 5 scRNA-seq dataset with repeated downsampling (10 cells/group/cell type/iteration, 100 iterations; *n* = 6 mice/group), median (IQR). Summary statistics provided as a Source data file. Macro macrophages, Peri pericytes, ABC arachnoid barrier cells, SMC smooth muscle cells, VLMC vascular leptomeningeal cells. **d** Volcano plot of pseudobulk DEGs in astrocytes Day 5, scRNA-seq dataset between FGF1 and vehicle. Blue, genes with FDR < 0.05; red, genes with FDR < 0.05 and |log_2_ fold change| > 0.25; green, genes with |log_2_ fold change| > 0.25; gray, genes below both thresholds. **e** Astrocytes WGCNA (Day 5; scRNA-seq) identified three significant upregulated (*sc-astro-M1*–*sc-astro-M3*; linear mixed-effects model; FDR < 0.05) and three downregulated (*sc-astro-M5*, *sc-astro-M6*, *sc-astro-M9*) modules in FGF1-treated mice, median (IQR). Lower panel: hypergeometric enrichment analysis of single-nucleus RNA-sequencing (snRNA-seq) and scRNA-seq astrocyte modules. Point size is scaled by −log_10_(*p* value) (Bonferroni adjusted; *n* = 6 mice/group). **f** Correlation of top 15 genes in modules *sc-astro-M1* and *sc-astro-M3*. **g** Representative images of immunoreactivity of aquaporin 4 (Aqp4; red) and glial fibrillary acid protein (GFAP; green) from coronal sections of hypothalamus from diabetic Lep^*ob/ob*^ mice 1 week after either vehicle or FGF1 icv injection. Lower panels: higher magnification of white inset. Scale bar 100 μm (top), 50 μm (lower). Pixel dimensions: 0.27 × 0.27 × 2.00 microns. Quantification: dots represent volume of Aqp4- or GFAP-positive voxels for individual animals (mean from 2 sections per animal, *n* = 4/group), mean ± SEM. Unpaired *t* test, two tailed, *p = 0.037, **p = 0.003 (not adjusted). Source data are provided as a Source data file. Genes mentioned in the text are labeled in red.

In response to various stimuli, the astrocyte phenotype can change rapidly and dramatically^13^. To date, neurotoxic, neuroprotective, pan-reactive, neuron-interactive, and neuronal activity-induced astrocyte phenotypes have been reported^25,31^. To test whether astrocyte responses on Day 1 or Day 5 after icv FGF1 treatment correspond to any of these phenotypes, we first performed WGCNA on astrocytes in the snRNA-seq dataset. Of the 7 identified modules, 3 were significantly increased on Day 1 (*sn-astro-M1*–*sn-astro-M3*) and 1 module was significantly increased on Day 5 (*sn-astro-M4*; Fig. 3a; linear mixed-effects models; FDR < 0.05; Supplementary Data 6). The 3 upregulated astrocyte modules on Day 1 enriched for markers of pan-reactive astrocytes, while *sn-astro-M2* and *sn-astro-M3* also enriched for markers of neuroprotective astrocytes (Fig. 3a). Although *sn-astro-M1* did not enrich for markers of either neurotoxic or neuroprotective astrocytes, its hub gene *Npas3* (Fig. 3b) is a transcription factor belonging to the bHLH-PAS superfamily known to be involved in FGF signaling, hypoxia, circadian rhythm, and metabolic pathways and has been proposed as a link between mental illness and metabolic disorders^32^.

In contrast, *sn-astro-M4*, which was upregulated on Day 5 post-FGF1 treatment, significantly enriched for neuron-interacting (NeurI) genes, suggestive of increased astrocyte–neuronal interaction. Among hub genes for *sn-astro-M4* was *Sparcl1*, a matricellular protein implicated in synaptic plasticity^33^, as well as genes encoding proteins involved in synaptic glutamate (*Ndrg2)* and potassium (*Kcnj10)* homeostasis (Fig. 3b). This module enriched for the terms “ammonium ion metabolic process,” “PPAR signaling pathway,” and “lipid metabolic process” (Supplementary Data 6), consistent with a model in which FGF1 helps to reduce neuronal stress^34^ through increased neuron–astrocyte metabolic coupling.

To confirm the snRNA-seq findings on Day 5 post-treatment and to gain more insight in the glial response to icv FGF1, we again utilized the larger scRNA-seq dataset. The relatively high number of resampled DEGs in astrocytes at Day 5 compared to the other cell glial cell types confirmed that, in these cells, the transcriptional response at Day 5 was more robust than at Day 1 (Fig. 3c). Pseudobulk analysis of astrocytes identified 186 DEGs on Day 5 (|log_2_ fold change (FC)| > 1; FDR < 0.05), of which 94 were upregulated and 92 downregulated, with *Gfap*, *Aqp4*, and *Vim* among the top upregulated genes (Fig. 3d; Supplementary Data 7). Using WGCNA, we identified 6 distinct astrocyte modules associated with FGF1 treatment on Day 5 (linear mixed-effects models; beta > 0.005; FDR < 0.05) (*sc-astro-M1*–*sc-astro-M6*; Fig. 3e; Supplementary Data 8), 3 of which were upregulated by icv FGF1. Hypergeometric enrichment revealed a significant overlap between *sn-astro-M4* and *sc-astro-M1* (10 genes in common) as well as with *sc-astro-M3* (7 genes in common; Fig. 3e). Module *sc-astro-M3* included genes involved in regulating homeostasis of both synaptic glutamate (*Slc1a2*, *Slc1a3)* and GABA (*Slc6a11*), as well as the major astrocyte water channel *Aqp4* (Fig. 3f). Module *sc-astro-M1* included genes regulating synapse formation (*Sparcl1*, *Sparc*), dendritic growth (*Psap*), and other neuroprotective functions (*Cp*e, *Clu*, *Plat*). These findings confirm the astrocyte response on Day 5 and are suggestive of multiple neuroprotective responses that range from changes in interstitial fluid homeostasis to direct regulation of synapse/dendrite formation (Supplementary Data 8).

To validate and extend these transcriptomic findings, we used immunohistochemistry (IHC) to determine whether expression of *Aqp4* (a shared upregulated gene at Day 5 in all astrocyte analyses and datasets) was increased at the protein level in astrocytes. We found that, in a separate cohort of diabetic Lep^*ob/ob*^ mice, Aqp4 protein content was increased 1 week after icv FGF1 injection, particularly in the mediobasal hypothalamus (MBH; Fig. 3g). We also detected a robust increase of glial fibrillary acidic protein (GFAP; encoded by *Gfap*) immunostaining in astrocytes 1 week following icv FGF1 injection, confirming the FGF1-induced astrocyte modules *sc-astro-M3* and *sn-astro-M2* containing *Gfap*. Combined with the robust, star-shaped astrocyte morphological change induced by FGF1 (Fig. 3g), these findings are suggestive of an astrocyte phenotype switch. Clarification of contribution made by these astrocyte responses to FGF1-induced glucose lowering is warranted.

### Unique temporal signatures in oligodendrocytes induced by icv FGF1

**Fig. 4:**
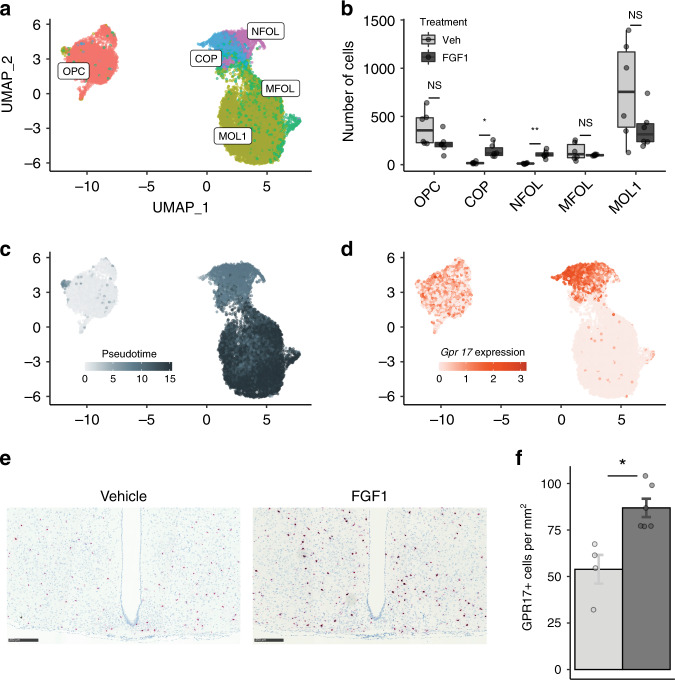
Icv FGF1 induces a population of differentiating oligodendrocytes. **a** UMAP clustering of cells in the oligodendrocyte lineage from the Day 5 scRNA-seq dataset. Cell type annotations were inferred from a published dataset^35^. **b** Dots represent mean number of cells per animal and lines represent mean ± SEM. Unpaired *t* test, two tailed, *p < 0.022, **p < 0.0074 (Bonferroni adjusted; *n* = 6 mice/group); ns not significant. Source data for each subcluster are provided as a Source data file. **c** UMAP of cells in the oligodendrocyte lineage labeled according to maturation in Monocle-derived pseudotime. **d** UMAP of cells expressing *Gpr17*, a maturing oligodendrocyte marker. **e** RNAScope detection of *Gpr17* expression in the hypothalamus of Lep^*ob/ob*^ mice 5 days after icv FGF1 or vehicle. **f** Quantification of *Gpr17*+ cells from the ARC area (2 sections per animal, icv vehicle (*n* = 4) and icv FGF1 (*n* = 5)), mean ± SEM. Unpaired *t* test, two tailed, *p = 0.005 (not adjusted). Source data are provided as a Source data file. NFOL newly formed oligodendrocyte, MFOL myelin-forming oligodendrocyte, MOL1 myelinating oligodendrocyte.

At a transcriptional level, oligodendrocytes displayed a distinct temporal response to FGF1. While evidence of minor immune activation was seen in mature oligodendrocytes on Day 1 (Supplementary Data 3), icv FGF1 had minimal effects on oligodendrocyte precursor cells (OPCs) at this early time point. By Day 5, however, our resampling test indicated a strong response in both oligodendrocytes and committed to differentiate oligodendrocyte precursors (COPs) (Fig. 3c). To better understand these responses, we extracted all oligodendrocyte lineage cells from our scRNA-seq dataset and mapped them to an existing dataset with a more detailed annotation of transcriptional oligodendrocyte heterogeneity^35^ (Fig. 4a). Retaining only cells that mapped with high confidence between datasets, we detected a significant increase in populations of both COPs and newly formed oligodendrocytes (NFOLs) in FGF1-treated mice (Fig. 4b) but not in mature oligodendrocytes.

We next used Monocle2^36^ to sort these cells along their likely developmental trajectories. The pseudotime trajectory confirmed the effect of icv FGF1 to increase the pool of cells at an intermediate pseudotime (Fig. 4c), corresponding to an accumulation of cells differentiating from OPCs toward mature oligodendrocytes. Using RNAScope to detect *Gpr17* expression as a proxy for COPs and NFOLs (Fig. 4d) in a separate cohort of Lep^*ob/ob*^ mice, we confirmed an increase in the number of these oligodendrocyte lineage cells in the ARC of icv FGF1-injected mice compared to vehicle-treated, PF controls (Fig. 4e), a response that appeared to extend beyond the MBH. Together, these results suggest that oligodendrocyte differentiation was induced by icv FGF1 in the hypothalamus of diabetic Lep^*ob/ob*^ mice. Although oligodendrocytes are well known to ensheath axons and provide metabolic support, and although glucose has been shown to be rapidly metabolized by oligodendrocytes^37^, we are unaware of evidence implicating oligodendrocyte lineage cells in the control of glucose homeostasis. Whether and how these cells might contribute to sustained FGF1-induced diabetes remission warrants additional study.

### Effect of FGF1 on neurons

**Fig. 5:**
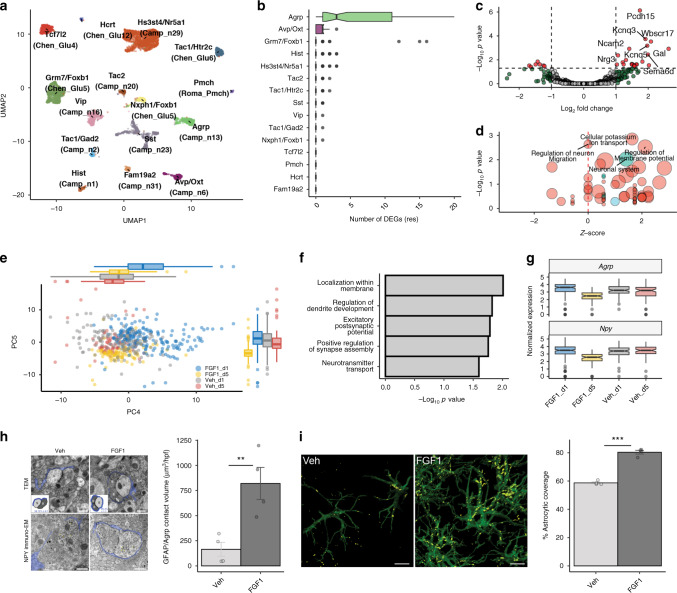
Effects of icv FGF1 on Agrp neurons. **a** UMAP clustering of neurons from the snRNA-seq dataset that confidently mapped to published datasets^16–18^. Original cell type annotations from the published datasets are indicated in brackets. **b** Numbers of DEGs for neuron subclusters identified using repeated downsampling (10 cells/group/cell type/iteration, 100 iterations; *n* = 3 mice/group), median (IQR). **c** Volcano plot of pseudobulk DEGs in Agrp neurons from the Day 1 snRNA-seq dataset between FGF1 and vehicle. Red, genes with FDR < 0.05 and |log_2_ fold change| > 0.25; green, genes with an |log_2_ fold change| > 0.25; gray, genes below both thresholds. **d** Gene Ontology (biological process; red) and REACTOME (blue) terms enriched for DEGs in Agrp neurons at Day 1. Circle size denotes gene overlap with the identified geneset. **e** Principal component analysis of Agrp neurons sampled at Days 1 and 5 (vehicle and FGF1) revealed strong separation from other groups at PC4 and PC5, respectively (*n* = 3 mice/group Day 1, *n* = 2 mice/group Day 5), median (IQR). **f** Gene Ontology term (biological processes) of highly loading genes on PC5. **g** Normalized expression of *Agrp* and *Npy* in Agrp neurons across the 4 groups (FGF1, Days 1 and 5; vehicle, Days 1 and 5; *n* = 3 mice/group Day 1, *n* = 2 mice/group Day 5). **h** Top panel: representative electron microscopic images of coverage of synapses between axonal boutons (B) and dendritic spines (S) by peri-synaptic astrocytic in the ARC area of Lep^*ob/ob*^ mice. Bottom panel: immunostaining for *Npy*. Scale bar 500 nm. Quantification: dots represent percentage of astrocytic coverage for individual animals (mean of 30 synapses per animal, *n* = 4/group), mean ± SEM. Unpaired *t* test, two tailed, ***p < 0.0001 (not adjusted). **i** Contacts between GFAP(+) astrocytes (green) and Agrp(+) neurons in the ARC area (equivalent to outline in **g**) 1 week following icv FGF1 or vehicle injection of Lep^*ob/ob*^. Yellow represents co-labeled GFAP-positive and Agrp-positive voxels. Scale bar 8 μm. Pixel dimensions: 0.057 × 0.057 × 0.500 microns. Quantification: dots represent mean contact volume over high power field (hpf: 116.5 × 116.5 microns) for individual animals (2 sections per animal, *n* = 4/group), mean ± SEM. Unpaired *t* test, two tailed, **p = 0.0093 (not adjusted). Source data and summary statistics are provided as a Source data file.

To confidently identify established hypothalamic neuronal subtypes in diabetic Lep^*ob/ob*^ mice, we propagated labels from published hypothalamic single-cell datasets^16–18^ (see “Methods”) and subclustered all confidently mapped cells into 15 hypothalamic neuron-specific cell populations (Fig. 5a). These neurons (11,986) were subclustered and manually annotated based on highly enriched genes and revealed a small cluster of Pomc-expressing cells (Supplementary Fig. 5a). Surprisingly, no neurons in our dataset were assigned with high confidence to previously characterized Pomc populations.

### Agouti-related peptide (Agrp) neurons are the most responsive neuronal subtype

To estimate which neuronal populations were most affected 1 day after icv FGF1 injection, we quantified the amount of DEGs for each neuronal subtype using a repeated downsampling approach^19^. This analysis revealed Agrp neurons to be the most FGF1-responsive neuronal population, with no other subsets (including Pomc neurons) having a median number of DEGs > 1 by Day 1 (Fig. 5b; Supplementary Fig. 5b). Utilizing the pseudobulk approach including all Agrp neurons from Day 1 (*n* = 313), we identified 47 DEGs (|log_2_ FC| > 1; FDR < 0.05), of which 37 were upregulated (Fig. 5c; Supplementary Data 9). To identify putative biological pathways activated by FGF1 in Agrp neurons, we subjected upregulated and downregulated DEGs identified in Agrp neurons to geneset enrichment analysis (GSEA) and found that, in Agrp neurons, FGF1 increases the expression of genes involved in both synapse assembly and regulation of membrane potential (Fig. 5d).

To investigate whether the response in Agrp neurons evolved over time, we compared Agrp neuron expression profiles between Day 1 and Day 5 after icv injection. To identify genes that drive variance in Agrp neurons across these two time points, we performed PC analysis (PCA) to identify those components which best separate the four groups (vehicle vs. FGF1, Day 1 vs. Day 5). In these neurons, the strongest differences in loading values were in PC4 and PC5, which, respectively, explained 1.9% and 1.7% of the variation in Agrp neurons and separated FGF1-Day 1 and FGF1-Day 5 Agrp neurons from the other Agrp neurons (Fig. 5e). The top loading genes on PC4 overlapped with genes identified as being significantly upregulated on Day 1, including *Pcdh15* and *Kcnq3*, suggesting that the effect of FGF1 to acutely induce expression of these genes in Agrp neurons had waned by Day 5 (Supplementary Data 10). Pathway enrichment analysis for negatively loading genes on PC5 (genes with increased expression in Agrp neurons in the FGF1 Day 5 group) was suggestive of changes in postsynaptic regulation and function (Fig. 5f). Furthermore, *Agrp* and *Npy* were the two most strongly positively loading genes on PC5, and expression levels of both genes were strongly reduced on Day 5 compared to all other groups (Fig. 5g). By comparison, neither *Agrp* nor *Npy* expression were reduced on Day 1 relative to PF, vehicle-treated controls, suggesting that the initial response of Agrp neurons to icv FGF1 injection (Day 1) involves alteration of genes implicated in the control of membrane potential, whereas the later response (Day 5) is predominated by decreased *Agrp* and *Npy* gene expression. Whether these findings are indicative of sustained, FGF1-induced Agrp neuron inhibition warrants additional study.

### FGF1 increases astrocytic contact with neurons

In light of both the important role played by astrocytes to modulate synaptic function^13–15^ and our evidence of a robust transcriptomic and histochemical response of astrocytes to FGF1, we next sought to test whether synaptic ensheathment by astrocyte processes is affected by FGF1. Electron microscopic analysis of the ARC area on samples obtained following icv injection of either vehicle or FGF1 in a separate cohort of Lep^*ob/ob*^ mice (*n* = 4/group) revealed an increase in coverage of synaptic boutons on dendritic spines by peri-synaptic astrocytic processes that included, but was not limited to, neurons positive for Neuropeptide Y (NPY; which is co-expressed with Agrp in ARC neurons; Fig. 5h).

Because these findings were associated with reduced *Agrp* and *Npy* expression levels, and because astrocytes are reported to modulate the tone of neurons in the melanocortin system^15^, we next asked whether physical interactions between astrocytes and Agrp neurons are induced by icv FGF1 injection. Consistent with this hypothesis, co-staining for GFAP and Argp performed 1 week following icv FGF1 (or vehicle) injection in a separate cohort of Lep^*ob/ob*^ mice reveals a marked increase of cellular contacts between GFAP(+) astrocytes and Agrp neurons induced by FGF1 in the ARC area (Fig. 5i). These findings point to a model in which the astrocyte response to FGF1 involves changes in synaptic function within hypothalamic neurocircuits relevant to glucose homeostasis. Such an effect need not be limited to Argp neurons, and further studies are warranted to determine whether and how enhanced glia–neuron interactions in the hypothalamus might contribute to the sustained antidiabetic action of FGF1.

### Dependence of FGF1-induced diabetes remission on melanocortin receptor signaling

**Fig. 6:**
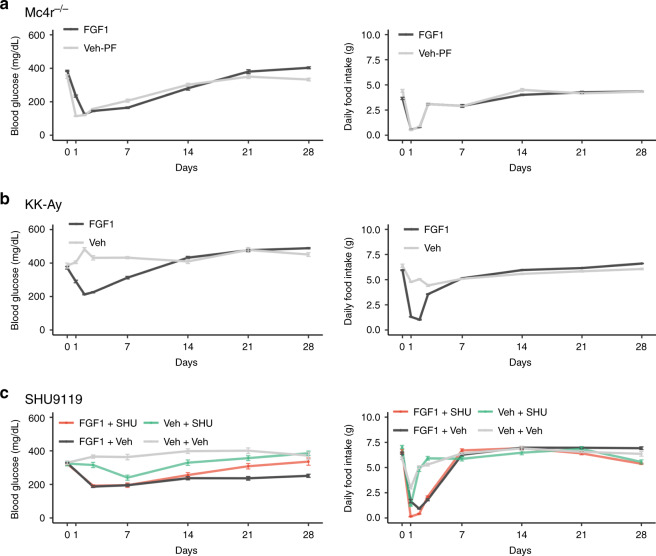
FGF1-induced sustained diabetes remission is melanocortin dependent. **a** Blood glucose levels and food intake in diabetic Mc4r^*−/−*^ (Mc4r knockout) mice injected with icv FGF1 (3 µg) or vehicle (*n* = 8/group). **b** Blood glucose levels and food intake in agouti (KK-A^*y*^) mice icv injected with FGF1 (3 µg) (*n* = 12) or vehicle (*n* = 9). Vehicle-injected mice were not pair-fed to the intake of mice receiving FGF1. **c** Blood glucose levels and food intake of diabetic Lep^*ob/ob*^ mice treated with either a single icv injection of FGF1 (3 µg) or vehicle, followed immediately by continuous icv infusion of either vehicle or the Mc4r antagonist SHU9119 (5 nmol/day) for 28 days. Veh+Veh (*n* = 8), FGF1+Veh (*n* = 9), Veh+SHU (*n* = 6), FGF1+SHU (*n* = 8). Data are represented as mean ± SEM, source data are provided as a Source data file.

Reduced melanocortin signaling is linked to diabetes pathogenesis in both rodents and humans^38–41^. Because Agrp neurons are the most responsive neurons after FGF1 injection, and since these neurons function as endogenous inhibitors of melanocortin signaling, we next asked whether sustained antidiabetic action of FGF1 depends on intact melanocortin signaling. To this end, we tested whether either mice lacking the melanocortin 4 receptor (Mc4r; Mc4r^*−/−*^) or agouti (KK-A^*y*^) mice (in which obesity and T2D (depending on the background strain) result from ectopic overexpression of Agouti, an Agrp homolog^42^) are resistant to the sustained antidiabetic response to icv FGF1 injection. We found that, whereas both Mc4r knockout and KK-A^*y*^ mice exhibited the rapid reduction of food intake and BG levels typical of Lep^*ob/ob*^ mice following icv injection of FGF1 (3 µg)^4^ (Figs. 1a and 6a, b), BG levels returned to baseline within 10–14 days in both mouse strains, unlike the prolonged normoglycemia induced by icv FGF1 injection in Lep^*ob/ob*^ mice^4^. Whereas intact melanocortin signaling is not required for initial anorexia and glucose lowering, these findings suggest that it is required for the sustained antidiabetic action induced by icv FGF1 injection.

To test this hypothesis further, we generated four groups of Lep^*ob/ob*^ mice, each of which received two separate icv treatments: a single icv injection of either FGF1 (3 µg) or vehicle, followed immediately by continuous icv infusion of either vehicle or the Mc4r antagonist, SHU9119 (5 nmol/day) for 28 days. Whereas food intake, body weight (BW), and BG levels did not change in icv vehicle-injected mice, regardless of whether they also received continuous icv infusion of vehicle or SHU9119 (Fig. 6c; Supplementary Fig. 6), icv FGF1 injection elicited both the expected initial reduction of food intake and the long-lived normalization of glycemia when it was coupled to continuous icv infusion of vehicle. When icv FGF1 injection was combined with continuous SHU9119 infusion, however, remission of hyperglycemia was not sustained even though the transient effects of icv FGF1 to reduce food intake, BW, and BG levels remained intact (Fig. 6c; Supplementary Fig. 6). Statistical analysis (Supplementary Notes) confirmed that, whereas icv SHU9119 infusion had no effect on transient reductions of food intake and glycemia induced by FGF1, it fully blocked sustained diabetes remission (Chi square (3) = 10.3, *p* = 0.016 (not adjusted)). These findings collectively demonstrate that, although increased melanocortin signaling is not required for the acute, transient effects of icv FGF1 injection on energy balance and glycemia, it is required for sustained diabetes remission.

### Long-term hypothalamic changes induced by icv FGF1

**Fig. 7:**
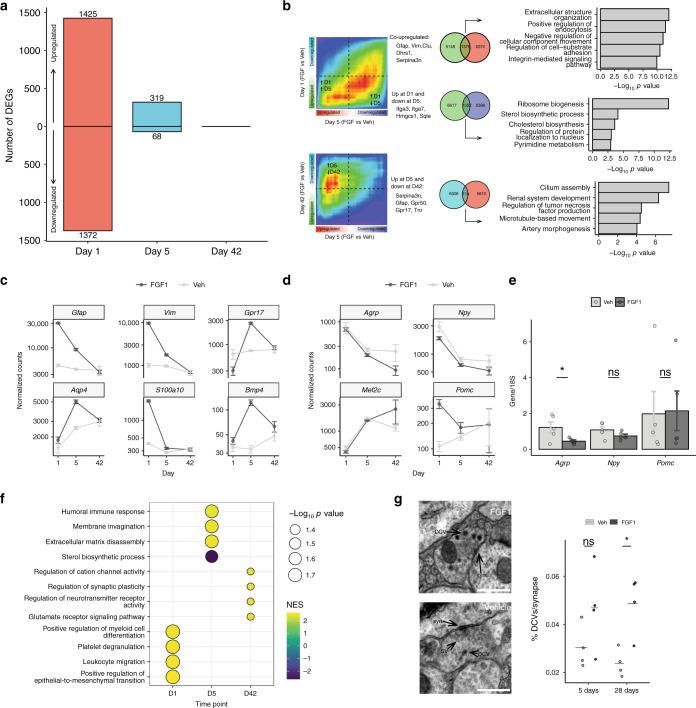
FGF1-induced long-term changes in the hypothalamus. **a** Number of DEGs (|log_2_ fold change| > 0.5; FDR < 0.05) from bulk RNA-sequencing (bRNA-seq) of hypothalami from Lep^*ob/ob*^ mice injected with icv FGF1 or vehicle and harvested Days 1 (FGF1 *n* = 10, vehicle *n* = 4), 5 (FGF1 *n* = 9, vehicle *n* = 7), and 42 (*n* = 5 mice/group) after injection. Control vehicle mice were pair-fed to FGF1-injected mice. **b** Rank–rank hypergeometric overlap maps of the degree of overlapping genes between Day 1 and Day 5 (top) and between Day 5 and Day 42 (bottom). Venn diagrams and top overlapping genes (middle panel). Enrichment of Gene Ontology biological process terms of overlapping genes (right panel). **c** Normalized counts (mean ± SEM, *n* = 5 mice/group) of the selected glial genes from the bRNA-seq datasets Days 1, 5, and 42 after icv FGF1 or vehicle injection. **d** Normalized counts (mean ± SEM, *n* = 5 mice/group) of *Agrp*, *Npy*, *Mef2c*, and *Pomc* expression Days 1, 5, and 42 after icv FGF1 or vehicle injection. **e** Targeted relative gene expression of *Agrp*, *Npy*, and *Pomc*. Unpaired *t* test, two tailed, *p = 0.04 (not adjusted, *n* = 5 mice/group); ns not significant. Source data are provided as a Source data file. **f** Threshold-free geneset enrichment analysis (Gene Ontology biological processes terms) of DEGs identified in bRNA-seq data at Days 1, 5, and 42. Point size is scaled by −log_10_(*p* value) (Bonferroni adjusted) and is colored based on the normalized enrichment score (NES). **g** Representative electron microscopic images of synapses of Lep^*ob/ob*^ mice 5 days after icv injection of either vehicle or FGF1. syn synaptic active zone, DCV dense-core vesicle, SV small light-core vesicle. Scale bar 500 nm. Quantification: dots represent mean %DCV/synapse per mouse (25 synapses per animal, *n* = 4/group). Unpaired *t* test, two tailed, *p = 0.01 (not adjusted); ns not significant. Source data are provided as a Source data file. Data are presented as mean ± SEM.

To investigate the durability of transcriptional responses elicited by icv FGF1, we generated bRNA-seq datasets from hypothalami of independent cohorts of Lep^*ob/ob*^ mice on Days 1, 5, and 42 post-icv FGF1 injection and compared the results to PF, vehicle-treated controls. DEG analysis revealed profound transcriptional changes at Day 1 that dissipated by Day 42 (|log_2_ FC| > 0.5; FDR < 0.05) Fig. 7a–c; Supplementary Data 11). Using rank–rank hypergeometric overlap analysis (RRHO)^43^ to identify transcriptome-wide patterns across the three time points, we identified a robust overlap in genes that were upregulated both at Days 1 and 5 as well as genes that were upregulated at Day 1 and downregulated at Day 5. As expected, among the upregulated genes at both time points were *Gfap, Vim*, and *S100a10* (another glial marker gene) consistent with the snRNA-seq analyses of a shared reactive glia response, while *Aqp4* and *Gpr17* and *Bmp4* (a COP marker gene) were upregulated only at Day 5 likewise confirming the single-cell analyses (Fig. 7c). Pathway enrichment analysis of co-upregulated genes indicated extracellular matrix remodeling and integrin signaling. Likewise, genes upregulated at Day 1 and not coherently regulated at Day 5 enriched for ribosome biogenesis, mirroring the dissipating response seen in tanycytes and ependymal cells. The Day 5 vs. Day 42 comparison revealed a strong overlap between genes upregulated at Day 5 and downregulated at Day 42. These genes enriched for pathways indicative of glial activity (e.g., “cilium assembly” and “gliogenesis”), suggesting that most of the robust glial responses observed on Days 1 and 5 had waned by Day 42 (Fig. 7b).

However, *Agrp* expression remained significantly downregulated in FGF1-treated mice compared to controls on Day 42 (log_2_ FC = −1.33 ± 0.39; *p* = 6.0 × 10^−4^; Bonferroni significance threshold, *p* < 0.05/30). This analysis also detected *Pomc* expression at low levels at each of the 3 time points, with a significant increase detected 1 day after FGF1 treatment (log_2_ FC = −1.66 ± 0.46; *p* = 2.9*10^−4^; Bonferroni significance threshold, *p* < 0.05/30). While this finding raises the possibility of Pomc neuron activation as an early component of the neuronal response to FGF1, by Day 42 *Pomc* levels had returned to those of vehicle-treated controls. To validate these observations, we performed a targeted analysis using reverse transcription polymerase chain reaction (RT-PCR), which confirmed that, 42 days after a single icv injection of FGF1 in Lep^*ob/ob*^ mice, *Agrp* expression was indeed reduced compared to vehicle controls, albeit not significantly when adjusting for multiple testing (*p* = 0.042; Bonferroni significance threshold, *p* < 0.05/3). Expression of *Npy* mRNA showed a similar trend at this late time point, but the effect did not achieve statistical significance, while *Pomc* expression was unchanged (Fig. 7d, e).

Among the most upregulated genes on Day 42 in Lep^*ob/ob*^ mice receiving FGF1 was *Mef2c* (Fig. 7d), which encodes a transcription factor that influences synaptic plasticity^44^. Furthermore, threshold-free GSEA on the Day 42 data indicated increased activity in neuronal pathways including “long-term synaptic potentiation”, “glutamate receptor signaling” and “regulation of synaptic plasticity”, while genes involved in “regulation of transmission of nerve impulse” were decreased (Fig. 7f; Supplementary Data 12). Because long-term presynaptic silencing can cause synaptic accumulation of dense-core vesicles (DCVs; neuronal organelles that contain neuropeptides)^45,46^, we investigated whether synaptic content of DCVs is affected by icv FGF1 injection. Thus electron microscopy was employed to evaluate 110 synapses from the MBH of Lep^*ob/ob*^ mice (*n* = 4/group) that received icv injection of either FGF1 or vehicle and were sacrificed 28 days later. The average number of DCVs/synapse/mouse was increased in mice receiving FGF1 by 67% (*p* = 0.010; Fig. 7g). Taken together, these findings offer indirect support for a model in which FGF1 induces sustained changes in presynaptic plasticity (afferent and/or local inputs) in the ARC area that contribute to long-term changes in circuit computations involved in central control of glucose homeostasis. Based on this model, we speculate that *Mef2c* induction by FGF1 contributes to a sustained reduction in the excitability of hypothalamic neurocircuits that raise the BG level when activated, leading to synaptic DCV accumulation and potentially contributing to sustained amelioration of hyperglycemia. Future studies are warranted to investigate this model.

## Discussion

Until recently, the brain’s ability to ameliorate hyperglycemia in a sustained manner in diabetic animals was unrecognized. Based on the untapped therapeutic potential of such an effect, the current work was undertaken to identify and characterize the response of diverse hypothalamic cell types to FGF1, with the ultimate goal of linking these responses to the sustained antidiabetic action of this peptide. To this end, we combined an unbiased, discovery-based transcriptomics approach with histochemistry, electron microscopy, and in vivo pharmacological and physiological studies. We identified dramatic transcriptional and morphological changes in both tanycytes and astrocytes in the days following icv FGF1 injection. At the ultrastructural level, increased astrocyte coverage of synaptic boutons was detected on dendritic spines of ARC neurons, including Agrp neurons. The transcriptome of Agrp neurons was also strongly altered following icv FGF1 administration, including a sustained inhibition of both *Agrp* and *Npy* expression. These observations raise the possibility that Agrp neuron function is altered as a consequence of FGF1 action on glial cells, and additional work is warranted to asses this possibility. If so, we would infer that the sustained glucose-lowering effects of central FGF1 involve actions on one or more hypothalamic glial cell types that in turn alter glucoregulatory neurocircuit function in a highly durable manner. To our knowledge, such an effect has not previously been reported.

Of particular interest was the response of tanycytes, which line the 3V and project long filamentous processes into adjacent hypothalamic nuclei involved in glucose homeostasis. These cells have glucose-sensing properties and are implicated as participants in hypothalamic control of metabolism^9,10,28,47^. Among the canonical signal transduction pathways activated downstream of FGF receptor activation is the MAPK-ERK pathway, and we recently reported that icv FGF1 injection rapidly induces MAPK-ERK signaling in tanycytes and ependymal cells lining the 3V^5^. It is therefore unsurprising that, within 24 h of icv FGF1 injection, tanycytes and ependymal cells were enriched for co-regulated genes known to be involved in the ERK cascade (including *Spry2* and *Spred1*^12^). Moreover, since tanycytes are implicated in ATP-dependent brain glucose sensing^47^, it is of interest that genes involved in glycolysis (which generates cytosolic ATP) were also upregulated in tanycytes at Day 5. Additional study is warranted to ascertain the contribution of these robust tanycyte responses to FGF1-induced normalization of glucose homeostasis.

In astrocytes, the most consistently upregulated genes are involved in neuroprotection. These include *Aqp4*, *Sparcl1*, and the glutamate transporters *Slc1a2* and *Slc1a3*, as well as *Clu*, *Cpe*, and *Ntrk2*. The latter is of interest because it encodes TrkB, the receptor for brain-derived neurotrophic factor (BDNF). Like FGF1, BDNF administration can ameliorate hyperglycemia in mouse models of T2D whether administered peripherally^48^ or when given icv at lower doses^49^, and BDNF is also implicated as an axon guidance cue for ARC neurons innervating adjacent hypothalamic areas during postnatal development^50^. Upregulation of TrkB in astrocytes is therefore of interest as a potential way to influence glucoregulatory neurocircuit organization.

Astrocytes also provide essential homeostatic support to neurons via multiple mechanisms. Among these are peri-synaptic astrocyte processes (PAPs) which, together with the presynaptic and postsynaptic terminals, comprise the “tri-partite synapse”^13^. As PAPs are essential both to normal synaptic function and to neuroprotection following brain injury^25^, our finding that PAP coverage of dendritic spines on ARC neurons was increased 5 days after icv FGF1 injection identifies a potential mechanism whereby FGF1 influences synaptic function of hypothalamic neurons involved in glucose homeostasis. This possibility is further strengthened by our finding that genes involved in astrocyte–neuron crosstalk^31^ were also upregulated in astrocytes and that FGF1 markedly increased cellular contacts between astrocytes and Agrp neurons.

With the observations that (1) morphological changes of hypothalamic astrocytes (astrogliosis) induced by high-fat feeding^15,51^ are associated with altered activity of both Pomc and Agrp neurons^15^ and (2) impaired melanocortin signaling is implicated in the pathogenesis of T2D in both humans and rodent models^38–41^, we hypothesized that the melanocortin system is a target for sustained diabetes remission induced by FGF1. Consistent with this notion, we report that sustained diabetes remission is not observed when icv FGF1 is given to diabetic mice with genetic impairment of melanocortin signaling (agouti mice, Mc4r^*−/−*^ mice), nor is it observed when melanocortin signaling is blocked pharmacologically in Lep^*ob/ob*^ mice (by icv infusion of an Mc4r antagonist). Combined with evidence that this effect of melanocortin blockade was observed despite the fact that FGF1-induced anorexia was unaffected, these findings imply that, whereas the sustained antidiabetic action of FGF1 is melanocortin dependent, the transient anorexic response is not. Based on these collective findings, we hypothesize that the antidiabetic action of FGF1 involves robust effects on hypothalamic glial cell types that in turn trigger sustained changes in the melanocortin system and perhaps other, as yet unidentified, glucoregulatory neurocircuits.

One limitation of the current work is that we identified only a small subset of Pomc neurons, possibly owing to the marked reduction of *Pomc* gene expression characteristic of Lep^*ob/ob*^ mice^52,53^. Nevertheless, we did observe an increase of *Pomc* expression in the bRNA-seq data on Day 1. If this response was accompanied by increased α-melanocyte-stimulating hormone release and binding to Mc4r, the resultant increase of melanocortin signaling might have played a role in the response to FGF1, potentially augmented by concomitant Agrp neuron inhibition. Additional investigation into the roles played by Pomc and Agrp neurons in the sustained antidiabetic action of FGF1 will be of considerable interest.

Another limitation inherent in transcriptomics research is that induced genes may or may not be translated into protein. We addressed this issue by employing IHC, in situ hybridization, and electron microscopy, but additional work is needed to clarify the physiological relevance of many of the observed transcriptional responses. Outstanding questions include: Which transcriptomic responses and cell types are direct targets for the effect of FGF1 on the melanocortin system? Which are affected secondarily? How do these changes evolve over time? What other glucoregulatory neurocircuits might be similarly affected?; And, which of these cellular responses are relevant to how the brain senses the circulating glucose level? Studies to address these questions are an important priority.

That a single icv injection of FGF1 is sufficient to re-set the defended level of glycemia to normal in rodent models of T2D^4,5,11^ is a finding of substantial therapeutic relevance. Ongoing efforts to bridge the gap between diverse cellular responses to FGF1 and the associated normalization of glycemia may ultimately uncover novel strategies for achieving sustained diabetes remission.

## Methods

### Mice

All procedures were either performed in accordance with the NIH Guide for the Care and Use of Laboratory Animals and were approved by the Institutional Animal Care and Use Committee at the University of Washington, Novo Nordisk Research Center Seattle, and St. Joseph’s Hospital and Medical Center or performed with approved protocols from The Danish Animal Experiments Inspectorate permit number 2014–15–0201–00181 and the University of Copenhagen project number P16-122. Male, 6–8-week-old Lep^*ob/ob*^ (B6.Cg-Lep^*ob*^/J), 6-week-old KK-A^*y*^ (KK.Cg-*A*^*y*^/J), and Mc4r^*−/−*^ (B6; 129S4-*Mc4r*^*tm1lowl*^/J) mice were purchased from Jackson Laboratories (Bar Harbor, ME, USA) and 8-week-old Lep^*ob/ob*^ (B6.V-Lep^*ob*^/JRj) mice were purchased from Janvier Labs, France.

Surgerized animals were individually housed under specific pathogen-free conditions in a temperature-controlled room, with 75–80% humidity and with a 12:12 h light:dark cycle and provided with ad libitum access to water and Purina 5008 chow (Animal Specialties, Inc., CA, USA) or Altromin 1310 chow (Brogaarden, Denmark) unless otherwise stated. Mice were allowed to acclimatize at least 7 days before surgery. After surgery, Mc4r^*−/−*^ mice were allowed to acclimatize for 2 weeks and subsequently put on a high-fat, high-sucrose diet (F1850, Cholesterol (0.15%), Paste; Bio-Serv, NJ, USA) to induce hyperglycemia.

### Surgeries and icv injections

Cannulation of the lateral ventricle (LV; 26-ga, Plastics One, Roanoke, VA) were performed under isoflurane anesthesia in either Lep^*ob/ob*^ or KK-A^*y*^ mice, using the following stereotaxic coordinates: −0.7 mm posterior to bregma; 1.3 mm lateral, and 1.3 mm below the skull surface^4^. Animals received buprenorphine hydrochloride (Reckitt Benckiser Pharmaceuticals Inc., Richmond, VA) or Rimadyl (5 mg/kg, Pfizer, NY, USA) postoperatively and were allowed to recover for at least 7 days before study, while food intake and BW were recorded. Mice whose BW had not recovered 7 days after surgery were excluded from the study.

Mice were closely monitored to ensure matched BW and BG levels between study groups. Lep^*ob/ob*^, Mc4r^*−/−*^, and KK-A^*y*^ mice that did not exceed BG of 250 mg/dl for 3 consecutive days were not considered diabetic and were excluded from the study. Recombinant human FGF1 (hFGF1 from Novo Nordisk A/S, Denmark) was dissolved in phosphate-buffered saline (PBS) at a concentration of 1.5 μg/μl. Mice were injected with either hFGF1 or vehicle (PBS) over 60 s in a final volume of 2 μl using a 33-gauge needle extending 0.8 mm beyond the tip of the icv cannula. Food intake, BW, and BG were recorded daily for mice receiving hFGF1 for 5 days and vehicle control mice were pair-fed to the mean amount eaten by hFGF1-injected mice for 5 days.

To determine whether the effect of icv FGF1 to induce sustained remission of hyperglycemia in Lep^*ob/ob*^ mice is dependent on melanocortin signaling, we commenced infusion of either saline vehicle or the melanocortin receptor antagonist SHU9119 (SHU, Bachem Americas, Inc., CA) immediately after administering a single icv injection of either saline vehicle or recombinant mouse FGF1 (3 µg; Prospec, NJ, USA) in a volume of 2.5 µl to male Lep^*ob/ob*^ mice in a volume of 2.5 μl as follows. Thus four groups of animals were studied: Group 1: vehicle (saline) + vehicle, Group 2: vehicle + FGF1 (3 µg), Group 3: SHU (5 nmol) + FGF1, and Group 4: SHU + vehicle. Vehicle or SHU (5 nmol/day) was continuously administered to Groups 1–2 or Groups 3–4, respectively, for 4 weeks using an osmotic minipump (ALZET, DURECT Corporation, CA) that was connected to an icv injector (PlasticOne) with polyethylene tubing (Tygon) and subsequently implanted subcutaneously to enable direct infusion of either vehicle or SHU. The injector was inserted into the LV cannula and fixed with both instant and orthodontic adhesives immediately following a single icv injection of either vehicle or FGF1.

### Generation of single-nucleus suspensions

Hypothalamic punches (2 mm) (Day 1, *n* = 3/group; Day 5, *n* = 2/group) were rapidly extracted between 9 and 12 a.m., dissected, and then placed ventral surface up into a chilled stainless steel brain matrix. A single coronal section (1.5-mm thick) block was obtained between the rostral and the caudal ends of the Circle of Willis, followed by a hypothalamic punch (2 mm), which was immediately frozen on dry ice and stored at −80 °C until further processing. For the single-nuclei dissociation, frozen hypothalamic punches were recovered in 1 ml ice-cold EZ PREP buffer (Sigma) and homogenized using 2 ml glass dounce tissue grinders; 10 times with pastel A and 10 times with pastel B, followed by 5-min incubation on ice. Homogenate was transferred to clean tubes and centrifuged at 500 × *g* for 5 min at 4 °C. Supernatant was removed followed by an additional 5 min incubation on ice in 1 ml EZ PREP buffer and centrifuged at 500 × *g* for 5 min at 4 °C. The pellet was washed first in 1 ml PBS (Gibco) with 1.0% bovine serum albumin (BSA) and centrifuged at 500 × *g* for 5 min at 4 °C followed by a 500 ml PBS + 0.1% wash containing LIVE/DEAD stain (Life Technologies) to ease nuclei visualization. Debris and myelin were removed from the resuspended nuclei by a 1.80 M sucrose gradient centrifugation step according to the manufacturer’s protocol (Nuclei PURE Prep Isolation Kit, Sigma). Nuclei were subsequently washed in 1 ml PBS + 1.0% BSA, filtered through a 40-mm mesh (Falcon), centrifuged at 500 × *g* for 5 min at 4 °C, and resuspended in 50–150 ml PBS + 1.0% BSA. Nuclei concentration was estimated by counting on a hemocytometer (iChip) and kept on ice until single-cell encapsulation.

### Generation of single-cell suspensions

Mice (*n* = 6/per group) were euthanized and brains were rapidly extracted, cooled in ice-cold Dulbecco’s modified Eagle’s medium/F12 media (Gibco, Thermo Fisher Scientific) for 5 min. Dissection of the brain was performed as above followed by a triangular section of the MBH using a scalpel. Dissected tissue was digested using a Neural Tissue Dissociation Kit (Miltenyi Biotec, Bergisch Gladbach, Germany) with manual dissociation with the following modifications: sections were immediately placed in preheated (37 °C) enzyme mix 1 (1425 ml Buffer X + 37.5 ml Enzyme E) and incubated in closed tubes for 15 min at 37 °C under slow, continuous rotation. Then enzyme mix 2 (15 ml Buffer Y + 7.5 ml Enzyme A) was added followed by mechanically dissociated using a wide-tipped, fire-polished Pasteur pipette by pipetting up and down 10 times slowly. After a 10-min incubation under slow, continuous rotation, using a series of 2 fire-polished Pasteur pipettes with incrementally smaller openings the tissue was gently dissociated until no visible pieces were observed. Cell suspensions were centrifuged at 300 × *g* for 10 min at room temperature (RT) and resuspended in 500 ml Hanks’ Balanced Salt Solution (HBSS) (catalog # 14025092, Gibco, Thermo Fisher Scientific) and 0.04% (w/v) BSA (catalog # A9418, Sigma-Aldrich). Following an additional wash (centrifugation at 300 × *g* for 5 min and re-suspension in 100 µl HBSS + 0.04% BSA), cells were filtered through a 40-mm mesh, and 50 ml HBSS + 0.04% BSA was added through the filter and transferred to clean tubes. The cell suspension was centrifuged at 300 × *g* for 5 min and resuspended in 50–150 ml HBSS + 0.04% BSA. Cell concentrations were estimated on a NucleoCounter NC-3000 (Chemometec, Denmark) and kept on ice until single-cell encapsulation.

### Generation of bulk RNA-seq data

Following euthanasia, brains (Day 1, *n* = 4 vehicle; *n* = 10 FGF1; Day 5, *n* = 7 vehicle; *n* = 9 FGF1; Day 42, *n* = 42/group) were dissected as for snRNA-seq and immediately frozen on dry ice and stored at −80 °C until further processing. RNA was isolated using the Qiagen RNeasy Mini Kit (catalog # 74104), followed by high capacity cDNA reverse transcription (from Applied Biosystems, catalog # 4368813), and finally analysis was carried the using Agilent RNA 6000 Nano Kit. The RNA sample concentration was between 200 and 350 ng/μl, with 10–15 μg purified RNA from each individual sample. All RNA samples showed a good purity (A260/280 ratios are between 2.0 and 2.1), and Bioanalyzer results demonstrated a good quality of RNA for all samples (RIN numbers > 9.2). Sample mRNA libraries were prepared using the Illumina mRNA True Strand Kit (Covance Genomics Lab, Redmond, WA; Seattle Genomics Core, Seattle, WA) and sequenced on either an Illumina HiSeq 2500 system to generate 100 bp paired end libraries with 20–30 million reads (Covance Genomics Lab, Redmond, WA) or Illumina NovaSeq 6000 system on an S1 flow cell yielding approximately 35 million (2 × 50 bp) paired end reads.

### Single-cell RNA sequencing

Single-cell cDNA libraries were generated using the Chromium single-cell platform and the 3′ v2 Reagent Kit according to the manufacturer’s protocol (10× Genomics, USA). Single-cell libraries were sequenced on a NextSeq 500 platform (3 samples (whole single cells) and 2 samples (single nuclei) on one flow cell) to obtain 100- and 32-bp paired end reads using the following read length: read 1, 26 cycles, read 2, 98 cycles and i7 index, 8 cycles.

### Single-cell RNA-seq and single-nucleus RNA-seq data processing

Cell Ranger version 1.2 (10× Genomics, USA) was used to de-multiplex and quantify UMIs. A count matrix was generated for each sample with default parameters and genes were mapped to the mouse reference genome GRCm38 (mm10, part of the Cell Ranger software package; for the scRNA-seq dataset) or an adapted reference from mouse reference genome including introns (for the snRNA-seq dataset), following the steps outlined on the 10× Genomics website (https://support.10xgenomics.com/single-cell-gene-expression/software/pipelines/latest/advanced/references#premrna). Barcodes were filtered to include those with a total UMI count >10% of the 99th percentile of the expected recovered cells.

### Quality control of single-cell and single-nucleus expression data

*Scrublet*^54^ was run with default parameters to identify likely doublets (this step was omitted for the snRNA-seq data), which were subsequently removed from the expression matrix. Further processing was performed using the *Seurat*^55^ R package (version 2.3). Cells in which <400 or >4000 expressed genes were detected, as well as any cell with >20% (>5% for snRNA-seq) mitochondrial and/or ribosomal transcripts, were discarded. Genes detected in <10 cells (<5 cells in the snRNA-seq data) were also removed. Counts were normalized using the *SCtransform()* from Seurat. Variable genes were identified using the *FindVariableGenes()* function with default settings. Unwanted sources of variation, e.g., number of UMI and ribosomal and mitochondrial percentage, were regressed out using the *ScaleData()* function. PCs were found with variable genes using the *RunPCA()* function. The top 30 PCs were used to identify clusters and with the *FindClusters()* function at a resolution of 0.1.

### Single-nucleus RNA-seq data clustering

UMAP projection of snRNA-seq data revealed a strong batch effect between samples in this dataset. To identify conserved populations between all samples, sample datasets were integrated using the Seurat commands *FindIntegrationAnchors()* and *IntegrateData()*. Following integration, the data were processed as described above. Following identification of marker genes with the *FindMarkers()* function, literature was searched for cell-specific genes to determine cluster identity. At this low-resolution clustering, we removed ambiguously mapping cells by identifying cells with low silhouette scores <0 and removed them from the dataset (https://perslab.github.io/bentsen-rausch-2020/). Finally, the data were re-clustered to identify major cell populations.

### Single-cell RNA-seq data clustering and identification of cell types

The scRNA-seq data showed much reduced batch effect as compared to the snRNA-seq data, so the integration step was unnecessary. Following identification of marker genes with the *FindMarkers()* function, literature was searched for cell-specific genes to determine cluster identity. All neuronal lineage cells were removed from this dataset, as the quality was poor due to mouse age (myelination/neuron fragility). The final step was to further remove any artifacts/doublets, which had been missed in the initial round of processing. Each cell type underwent a round of iterative clustering as described above. Within each cell type, any cluster in which 50% of cells originated from a single sample was removed. The *FindMarkers()* function was used to identify markers of each subcluster, which were removed if enriched for marker genes of a different cell type. Finally, all remaining cells were merged to create the final dataset, and clustering was performed again with the same settings as above.

### Label projection

To identify canonical hypothalamic neuron populations, we downloaded three existing single-cell studies characterizing cell types in the MBH region. The raw data were processed with *Seurat* using default parameters and original labels were maintained. The projection was performed iteratively in that we first took the Campbell et al.^16^ data (GSE93374) and used the *FindTransferAnchors()* and *TransferData()* commands (default settings, 30 dimensions) to transfer labels to the query data. All cells that were assigned a label with >75% confidence were removed from the query dataset. We then performed label projection using the Chen et al.^18^ (GSE87544) and the Romanov et al.^17^ (GSE74672) datasets as described above. All mapped cells were then extracted and reclustered as described above. Clusters were annotated based on marker genes and the projected label that appeared most times in the identified cluster.

### Differential gene expression resampling

A strong driver of differential expression in single-cell data is the cell number. To control for this, our resampling procedure randomly selects ten cells per cluster per sample for pseudobulk analysis (see below). The test was performed 100 times per cluster to generate a distribution of DEGs. Bonferroni adjustment was used to account for multiple testing.

### Pseudobulk analysis

Pre-determined clusters were split by sample. Raw counts were summed across each gene per sample to generate a single column per sample. This matrix was then fed directly into DESeq2 tool (version 1.22.2)^56^ for standard processing.

### Pathway enrichment analysis for pseudobulk results

DEGs were tested for functional enrichment of GO (GO:BP category)^20^, REACTOME^21^, and KEGG^22^ pathways using the gProfiler R package^57^. gProfiler parameters were adjusted to indicate the use of an ordered query, limit testing to pathways containing between 10 and 300 genes, a minimum intersection size of 3 genes, and a strong hierarchical filtering of GO terms. A custom background was set for all analyses to include all genes that were used for DESeq2 analyses. A *z*-score was calculated for each term by calculating the number of upregulated minus downregulated genes divided by the square root of the number of genes that overlapped within a term.

### WGCNA and adjacency matrix calculation

The *WGCNA* package (version 1.66)^23^ implemented in R was used. Pearson correlation coefficient was used for the single-cell data and *signed network* parameter was used to compute the gene–gene adjacency matrix. Powers corresponding to the top 95th percentile of network connectivity or above were discarded and the lowest soft threshold power between 1 and 30 to achieve a scale-free topology *R*-squared fit of 0.8 was selected.

### WGCNA hierarchical clustering

The topological overlap matrices were converted to distance matrices, and the *hclust()* function was used with the *average* (clusters <3000 cells) or *complete* (clusters >3000 cells) method to cluster genes hierarchically. The *cutreeDynamic()* function was used with a *deepSplit* parameter of 2 (clusters >3000 cells) or 4 (clusters <3000 cells) and the *pamStage* parameter set to FALSE to carve the dendrogram into modules with at least 15 genes. The *mergeCloseModules()* function was used to compute module eigengenes, the vector of cell embeddings on the first PC of each module’s expression submatrix. The same function was used to merge modules, using a *cutHeight* of ≤0.2, corresponding to a Pearson correlation between module eigengenes of ≥0.8. The module eigengenes and expression matrices were used with the *signedKME()* function to compute gene–module Pearson correlations, or *kMEs*, a measure of how close each gene is to each module. Module treatment associations were tested using a linear mixed-effects model with the *nlme* R package^58^ (function *lme()*), using a random effect for each sample. Treatment and batch were modeled as fixed effects to control for variation between samples. Significance values are FDR corrected to account for multiple testing.

### Geneset enrichment analysis

Genes within each module were ordered based on their module membership (kME) and tested for functional enrichment of GO:BP terms^20^, REACTOME^21^, and KEGG^22^ pathways using the gProfiler R package^57^. gProfiler parameters were adjusted to indicate the use of an ordered query, limit testing to pathways containing between 10 and 300 genes, a minimum intersection size of 3 genes, and a strong hierarchical filtering of GO terms. A custom background was set for all single-cell analyses to include only the 5000 most variable genes, which were used for WGCNA-based analyses.

### Tanycyte analysis

Labels were propagated from the Campbell et al. dataset^16^ (GSE93374) using the *Seurat* (version 3.0) *SCTintegration()* pipeline with default settings and 30 dimensions. All cells that were assigned a label with >50% confidence were further analyzed. PCA was performed on all tanycytes using the *RunPCA()* function in Seurat. Upon identification of a gene expression continuum in PC1/PC2, the *princurve* R package^59^ was used with default settings to fit a principal curve to the data. Each cell was assigned a lambda value along the principal curve to be used as a measure of pseudoventricle height. To calculate the effect of FGF1 on module expression over pseudoventricle height, module expression was binned with a width of 1. Average expression and SEM was calculated at each pseudoventricle value. Linear regression was performed to identify significant changes in expression.

### Astrocyte analysis

To better characterize genesets induced in astrocytes by FGF1, hypergeometric enrichment was performed to test whether the genesets enriched for markers of astrocytes in middle cerebral artery occlusion (MCAO)/lipopolysaccharide (LPS) mice or astrocytes co-cultured with neurons. Prior to the analysis, the Zamanian et al.^25^ (GSE35338) and Hasel et al.^31^ (E-MTAB-5514) datasets were downloaded and DEG analysis was performed. Genesets were constructed by identifying any gene with a log_2_ FC > 2 and FDR < 0.05 (omitted for the Zamanian et al.^25^ dataset) in any of the conditions described. Any genes shared between LPS and MCAO at Day 1 were assigned to the PAN-reactive geneset. Furthermore, any gene found to overlap between LPS and MCAO was removed from the geneset. All *p* values were FDR corrected.

### Oligodendrocyte analysis

Labels were propagated from Marques et al.^35^ (GSE75330) using the *Seurat* pipeline with default settings and 30 dimensions. All cells which were assigned a label with >50% confidence were further analyzed. Shifts in cell type abundance across conditions were tested using the Student’s *t* test. To further analyze the effects of FGF1 on oligodendrocytes, a pseudo-developmental timeline of oligodendrocyte differentiation was built with Monocle2^36^. Cells were ordered based on genes that differ between clusters, a procedure referred to as *dpFeature*. tSNE dimensional reduction was performed using two PCs. Cells were then clustered in this with the *clusterCells()* command with a local density (*p* value) value of 150 and a nearest distance of a cell (*delta*) value of 15. DEGs between clusters were identified with the *differentialGeneTest()* command. The top 1000 DEGs were extracted for cell ordering. The pseudotime axis of differentiation was determined based on the expression of previously established markers.

### Bulk RNA-seq data processing and differential gene expression analysis

The RNA-seq sequences were aligned to the *Mus musculus* reference genome build Grch38 (release 77) using the STAR aligner^60^. The resulting count data were quantile normalized and used for downstream analysis. The resulting count matrix were analyzed with DESeq2 to perform count normalization and differential expression analysis between treatment groups.

### RRHO analysis

Gene lists from each time point were ranked by *t*-statistics. RRHO was then used to evaluate the transcriptome-wide overlap of these lists using the web application at https://systems.crump.ucla.edu/rankrank/rankranksimple.php^43^.

### IHC, microscopy, and image analysis

IHC of diabetic Lep^*ob/ob*^ mice treated with icv FGF1 (3 μg) or vehicle were perfused 7 days after injection with saline and 4% paraformaldehyde (PFA), post-fixed overnight at 4 °C, and then sectioned using a vibratome (50 µm). For immunostaining, sections were incubated in primary and secondary antibodies in PBS/0.5% Triton-X100 and 5% normal goat serum for 24–48 h at 4 °C. Primary antibodies were chicken anti-vimentin (1:500, EMD Millipore AB5733), mouse anti-GFAP (1:500, EMD Millipore MAB3402), rabbit anti-Aquaporin 4 (1:200, EMD Millipore AB3594), and rabbit anti-Agrp (1:1000, Phoenix Pharmaceuticals, Inc., 003–53). Secondary antibodies were goat anti-chicken 555 (1:500, Invitrogen, A11039), goat anti-mouse Alexa 488 (1:500; Invitrogen, A28175), and donkey anti-rabbit Alexa 594 (1:500; Invitrogen, A-21207). Confocal images were taken on a Leica SPE Confocal Microscope. To quantify the amount of labeling with different antibodies, we used high-resolution confocal *z*-stacks to reconstruct the ventromedial ARC region in two coronal sections containing the ME (bregma −1.7 and −2.0). For images capturing a wide area of the MBH including the third ventricle, we used a ×20 ACS APO multi-immersion objective (NA 0.6). For images taken at higher power to examine contacts between GFAP- and Agrp-positive processes in the ARC, we used a ×63 ACS APO oil immersion objective (NA 1.3). Imaris image analysis software version 9.1.0 (Bitplane) was then used to determine the volume of label-positive voxels (summation of voxels with intensity levels above a fixed threshold designated as positive and kept consistent across all images across all groups) throughout the region of interest (ROI; i.e., ARC) and compared across study groups. For quantification of GFAP–Agrp contacts, we determined the volume of co-labeled voxels expressing both GFAP and Agrp at intensities above the threshold value in high-resolution confocal stacks of the mediobasal ARC. While there are no cells that co-express both of these markers, the co-labeling of individual voxels was used as a measure of proximity between GFAP- and Agrp-expressing cell types.

### Electron microscopy

Two cohorts of Lep^*ob/ob*^ mice were icv injected into the LV with either hFGF1 or vehicle (pair-fed to the hFGF1-injected mice) as described above. One cohort was sacrificed 5 days postinjection, and the second cohort was sacrificed 28 days postinjection. Mice were anesthetized with ketamine and xylazine and transcardially perfused with 0.1 M PBS followed by 2% PFA and 2.5% glutaraldehyde in 0.1 M PB (pH 7.4), following which the brains were post-fixed in the same fixative overnight at 4 °C. The brains were then washed 6× with 0.1 M PB at 4 °C, serially sectioned at 200 μm, and washed overnight in fresh 0.1 M PB. Sections were further post-fixed in 2% osmium tetroxide (Electron Microscopy Sciences) in 0.1 M PB for 1 h at RT in the dark and washed 3× with cold d-H_2_O. Sections were then dehydrated 1× in 25% ethanol, 1× in 50% ethanol, and 1 ×  70% ethanol at 4 °C, followed by an incubation in 2% uranyl acetate (Electron Microscopy Sciences) dissolved in 70% ethanol (Electron Microscopy Sciences) in the dark for 3 h at 4 °C. Sections were then washed 1× in 70% ethanol, 3× in 95% ethanol, and 3× in 100% ethanol, then washed 2× with propylene oxide at RT and embedded in Durcupan (ACM; Fluka, Germany). Ultrathin sections (60–70 nm) were obtained with a diamond knife in a UC7 ultramicrotome (Leica, Germany) and stained with lead citrate (Reynolds’ solution)^61^. For pre-embedding NPY immunogold staining, mice were fixed with 4% PFA in 0.1 M PB (pH 7.4) as described above. Brain sections were incubated with rabbit anti-NPY (1:1500, Abcam, ab30914) for 72 h at 4 °C and incubated with gold-conjugated goat anti-rabbit antibody overnight followed by silver enhancement^62^. MBH astrocyte coverage of synaptic boutons and synapses were then imaged at 80 kV on a FEI Tecnai G^2^ Spirit transmission electron microscope (FEI Europe, Netherlands) equipped with a Morada CCD digital camera (Olympus Soft Image Solutions GmbH, Münster, Germany). Astrocyte coverage was determined by quantitation of the synapse surface in contact with electron-lucent astroglial expansions relative to the total perimeter of the synapse. For each synapse, the number of DCVs and the total number of synaptic vesicles were quantified using Fiji’s (version 1.51j8) Cell Counter plugin^63^.

### RNAScope

In a separate cohort of Lep^*ob/ob*^ mice, hFGF1 or vehicle (pair-fed to the hFGF1-injected mice) was icv injected as described above. After 5 days, the mice were anesthetized with ketamine and xylazine and transcardially perfused with saline followed by fresh 10% neutral buffered formalin. Dissected brains were post-fixed at RT for 8–12 h, then cut into 3 slabs, and further post-fixed for 16–32 h in total. Slabs were followed by ethanol for 2–4 days and subsequently embedded in paraffin. Sections were cut at 3–5 µm and single in situ hybridization (ISH) was performed with the RNAscope® 2.5 VS Reagent Kit (catalog # 323250, Advanced Cell Diagnostics, Newark, CA) on a Ventana Discovery Ultra platform (Roche Diagnostics, Penzberg, Germany). Pretreatment for ISH was 24 min target retrieval and 16 min protease treatment. The probe applied targets mouse Gpr17 mRNA (catalog # 318139), and signal was detected using the Fast Red Detection Kit (catalog # 8127166001, Roche Diagnostics, Penzberg, Germany). For image analysis, the HALO ISH-IHC version 1.3 app (Indica Labs, Albuquerque, NM) was used on two adjacent sections from the ARC region from each brain. In brief, the area was drawn as an ROI on all images by an observer blinded to treatment groups. Then all GPR17-positive cells were automatically detected and counted using threshold settings for the HALO app that detected all positive cells in all samples, with minimal background signal.

### Relative gene expression (RT-PCR)

Total RNA was extracted from hypothalami using TRI Reagent (Sigma-Aldrich) and NucleoSpin RNA (Thermo Fisher Scientific). Levels of hypothalamic transcripts were quantified by real-time PCR (ABI Prism 7900HT; Applied Biosystems) using SYBR Green (Applied Biosystems), and results were normalized to the housekeeping gene 18s. For comparative analysis, RNA ratios of the treatment group were normalized to the icv Veh control group.

### Other statistical analyses

Statistical analyses were performed using Prism 7 (Graph Pad) and R (R Core Team, 2018. https://www.R-project.org/). Data are expressed as dot plots representing data from individual animals or as mean ± SEM unless otherwise indicated. Descriptive statistics and two-tailed *t* tests were computed in GraphPad Prism 7 and probability values <0.05 were considered statistically significant. To assess differences of BG across the 4 study groups over the entire 35-day treatment period shown in Fig. 6c, individual time series data for BG were summarized as the area under the curve (AUC; units of (mg/dl) × days) using the trapezoidal rule. AUC data were analyzed and adjusted for baseline BG using analysis of variance (ANOVA). ANOVA was also used to assess BG on the final (35th) day of treatment. To specifically assess the effect of time on group BG responses from Day 3 to Day 35 across the four groups, the time series data in mg/dl were analyzed using the linear mixed model approach. Repeated measurements were accounted for using subject-level random intercepts and slopes^64^, while the fixed effects were group, time, the group × time interaction, and baseline BG. Food intake was similarly analyzed both as AUC (using ANOVA) and as a function of time (using mixed model analysis). Models were adjusted for baseline food intake. For analyses performed in R, ANOVAs were performed using the linear model (lm) procedure, mixed model analyses were performed using the linear mixed-effects (lme4) procedure, and group contrasts were tested using the generalized linear hypothesis test in the *multcomp* package.

### Reporting summary

Further information on research design is available in the Nature Research Reporting Summary linked to this article.

## Supplementary information

Supplementary Information

Description of Additional Supplementary Files

Supplementary Data 1

Supplementary Data 2

Supplementary Data 3

Supplementary Data 4

Supplementary Data 5

Supplementary Data 6

Supplementary Data 7

Supplementary Data 8

Supplementary Data 9

Supplementary Data 10

Supplementary Data 11

Supplementary Data 12

Reporting Summary

## Data Availability

All RNA-seq data generated in this study (snRNA-seq, scRNA-seq and bulk RNA-seq) are available through the NCBI Gene Expression Omnibus (GEO) under Super Series accession number GSE153551. This study also used publicly available datasets, including “A Molecular Census of Arcuate Hypothalamus and Median Eminence Cell Types” (GSE93374)^16^, “Single-cell RNA-seq of mouse hypothalamus” (GSE74672)^17^, “Single-cell RNA-seq reveals hypothalamic cell diversity” (GSE87544)^18^, “Expression data from reactive astrocytes purified from young adult mouse brains” (GSE35338)^25^, “RNA-seq analysis of single cells of the oligodendrocyte lineage from nine distinct regions of the anterior-posterior and dorsal-ventral axis of the mouse juvenile central nervous system” (GSE75330)^35^, and “RNA-seq of mouse astrocytes in monoculture and co-cultured with rat neurons (both inactive and active)” (E-MTAB-5514)^31^. The authors declare that all the data supporting the findings of this study are available within the article and its supplementary information files or from the corresponding authors upon request. Images are available upon request from the corresponding authors. Source data are provided with this paper.

## Source data

Source Data

## Code availability

Codes are made available through https://perslab.github.io/bentsen-rausch-2020/. Names, references, and version numbers of all software packages used are stated in the “Methods” section.
