# Supplementary material for: Transcriptomic analysis links diverse hypothalamic cell types to fibroblast growth factor 1-induced sustained diabetes remission
Source: Nat Commun. 2020 Sep 7;11:4458. doi: 10.1038/s41467-020-17720-5 (PMC7477234; doi:10.1038/s41467-020-17720-5)
Supplement: Supplementary file 1 — Supplementary Information [file 41467_2020_17720_MOESM1_ESM.pdf]

Supplementary Information

Transcriptomic analysis links diverse hypothalamic cell types to fibroblast growth factor 1-induced Sustained Diabetes Remission

Bentsen MA, Rausch DM *et al.*

Supplementary Figures

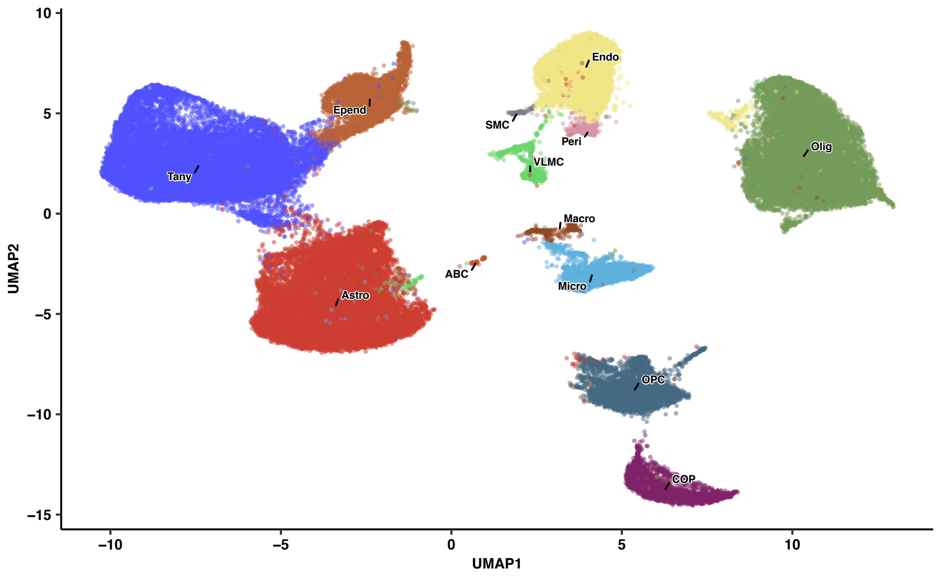

Supplementary Figure 1. Uniform manifold approximation and projection (UMAP) clustering of hypothalamic non-neuronal cells from Day 5 single-cell RNA-sequencing dataset; Epend, ependymal cells; Tany, tanycytes; Astro, astrocytes; OPC, oligodendrocyte precursor cell; COP, committed to differentiate oligodendrocyte; VLMC, vascular leptomeningeal cell; Endo, endothelial cells; Micro, microglia; Olig, oligodendrocytes; Macro, macroglia; Peri, pericytes; SMC, smooth muscle cells.

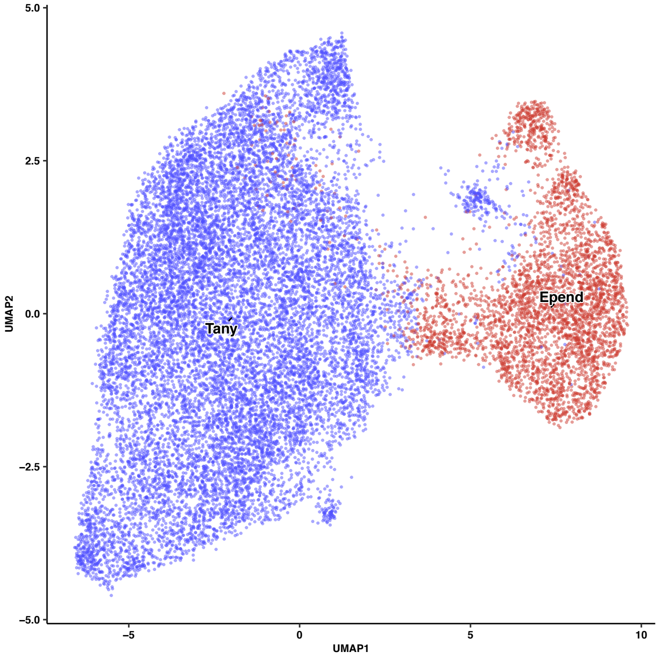

Supplementary Figure 2. Uniform manifold approximation and projection (UMAP) clustering of ependymal cells (red) and tanycytes (blue).

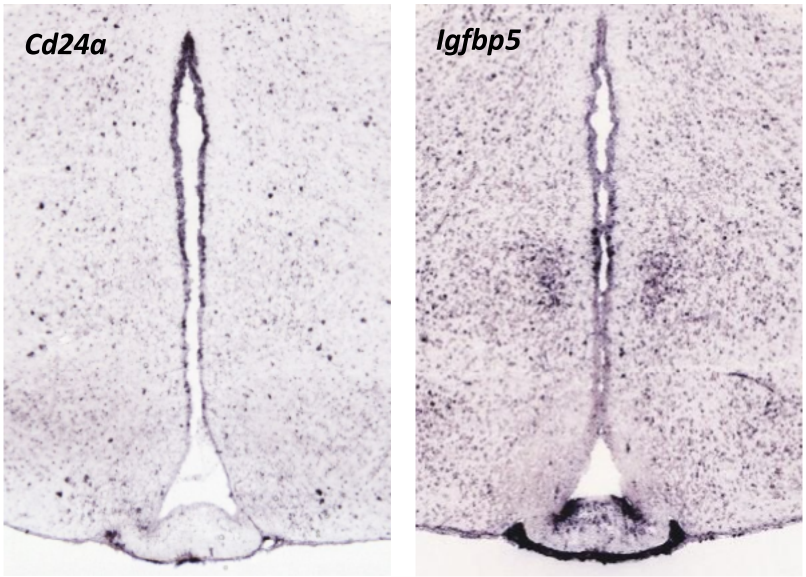

Supplementary Figure 3. Expression of *Cd24a* and *Igfbp5* shown by in situ hybridization, coronal section of the mediobasal hypothalamus (Image credit: Allen Institute. © 2004 Allen Institute for Brain Science. Allen Mouse Brain Atlas. Available from: *Cd24a*: https://mouse.brain-map.org/gene/show/12269; *Igfbp5*: https://mouse.brain-map.org/gene/show/15784)[1].

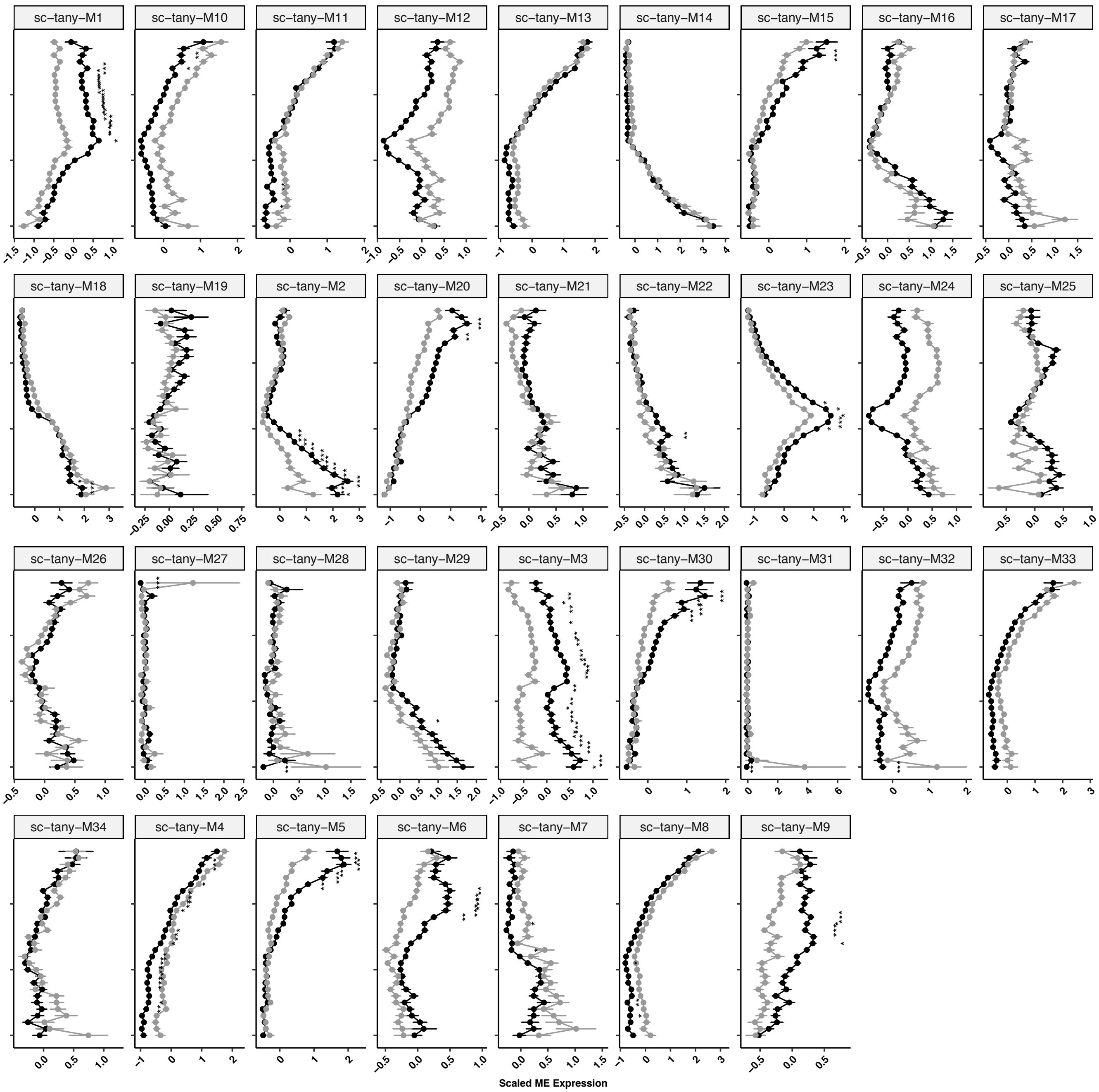

Supplementary Figure 4. Scaled module expression of tanycyte modules in FGF1 treated (black) and vehicle (grey) along the pseudoventricle trajectory constructed based on the single-cell RNA-sequencing data (linear mixed-effects model; bin size of 1; ±SEM; *p<0.05, **p<0.01, ***p<0.001) (Bonferroni adjusted), n=6 mice/group. Exact adjusted *p*-values are provided in Supplementary Table 5. ME, module eigengene.

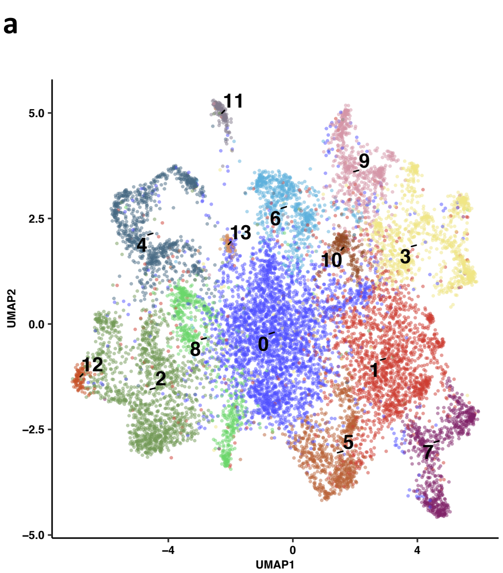

1	*Nrx3*
2	*Ebf1*
3	*Gal*
4	*Oxt*
5	*Meis2*
6	*Cacna2d1*
7	*Zfpm2*
8	*Nxph1*
9	*Pomc*
10	*Satb2*
11	*Sphkap*
12	*Trh*
13	*Itih3*

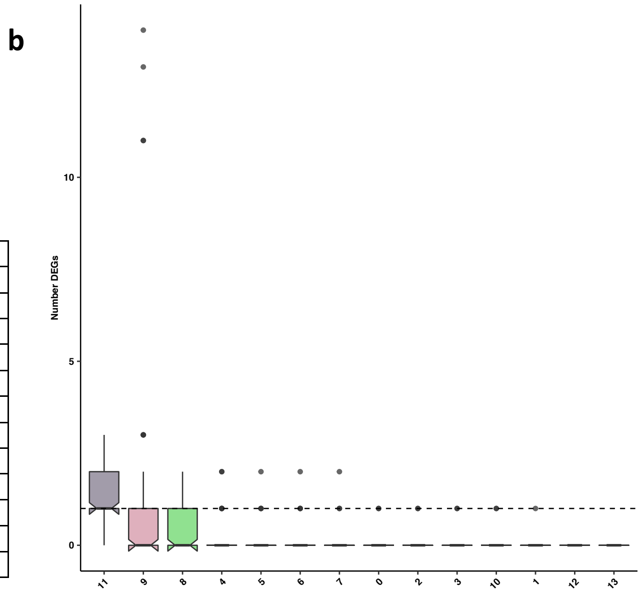

Supplementary Figure 5. a, Uniform manifold approximation and projection (UMAP) clustering of neurons from single-nucleus RNA-sequencing dataset that did not confidently map to cell populations reported in Campbell *et al.*[2], Romanov *et al.*[3], and Chen *et al.*[4]. Top differentially expressed genes (DEGs) within each cluster compared to all other unmapped neurons in table. **b,** Identification of number of DEGs for unmapped neuron subclusters with a repeated downsampling (10 cells/group/cell type/iteration, 100 iterations; n=3 mice/group. Data is presented as median and interquartile range. Summary statistics is provided as a Source Data file.

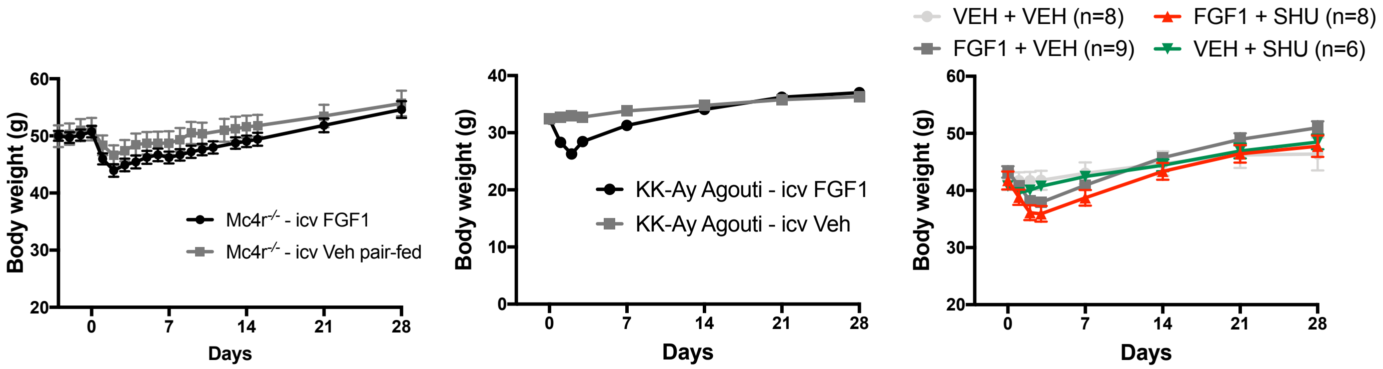

Supplementary Figure 6. Body weight of Mc4r$^{-/-}$ mice (n=8/group), KK-A^{y} mice (FGF1 n=12, vehicle n=9) and Lep$^{ob/ob}$ mice treated with either a single intracerebroventricular (icv) injection of FGF1 (3 µg) or vehicle, followed immediately by continuous icv infusion of either vehicle or the Mc4r antagonist, SHU9119 (5 nmol/day) for 28 days, Veh+Veh (n=8), FGF1+Veh (n=9), Veh+SHU (n=6),

FGF1+SHU (n=8). Data are represented as mean±SEM and source data are provided as a Source Data file.

Supplementary Notes

Tanycyte activation is sustained through Day 5 following icv FGF1 injection

Expression of the module *sc-tany-M2* was specifically increased in the ventral part of the pseudoventricle (main text **Fig. 2f**), corresponding to β2-tanycytes which form the interface between the fenestrated capillaries in the ME, a circumventricular organ strongly implicated in metabolic homeostasis, and cerebrospinal fluid in the 3V. This module included immediate-early genes *Fos*, *Egr1* and *Jun*, and the transcription factor *Hes1*. The most enriched GO term for this module was 'labyrinthine layer development', a process that integrates the fetus' and the mother's blood vessels in the placenta[5]. These results support the notion that β2-tanycytes have stem cell-like properties[6] and suggest that icv FGF1 triggers a gene program in the ME that is reminiscent of blood barrier formation in the placenta. We found that *sc-tany-M3* showed a significant FGF1-induced increase in expression across nearly the entire pseudoventricle (main text **Fig. 2f**). This module was comprised largely of genes encoding ribosomal proteins involved in protein synthesis, mirroring the response of tanycytes on Day 1. One module, *sc-tany-M4*, was significantly reduced compared to vehicle. Pathway analysis implicates this gene module in fatty acid metabolism (Supplementary Table 5). While both modules *sc-tany-M5* and *sc-tany-M6* were expressed dorsally along the pseudoventricle, they were not overlapping. The hub genes for these modules, *Cd24a* and *Igfbp5*, respectively, have been identified in progenitor cells in the ventricular-subventricular zone[7] and *in situ* hybridization confirmed expression of these genes in cells lining the 3V[1] (Supplementary Fig. 3). Module *sc-tany-M23* displayed a very distinct expression pattern along the ventricle characteristic of α2-tanycytes. This module contained several genes of the peroxiredoxins family which have antioxidant properties,

suggesting a role for tanycytes in the hypothalamic response to oxidative stress that is upregulated by FGF1. Interestingly, oxidative stress is reported to be an aberrant hypothalamic manifestation of obesity[8], and tanycyte processes project widely into adjacent hypothalamic areas implicated in glucose homeostasis and nutrient sensing, including the median eminence (ME), arcuate nucleus (ARC), ventromedial hypothalamus (VMH) and dorsomedial hypothalamus (DMH). Furthermore, increasing evidence suggest tanycytes themselves are nutritional sensors[9,10].

Ependymal cell responses Day 5 following icv FGF1 injection

To identify sets of co-regulated genes, we applied WGCNA to ependymal cells separately from the larger single cell RNA-seq data set and identified 3 modules that were significantly upregulated by FGF1 (linear mixed-effects model; FDR<0.05; Supplementary Table 4). Module *sc-epen-M1* resembled module *sn-glia-M4*, which was specifically upregulated in ependymal cells Day 5 in the snRNA-seq analysis. Consistent with their known function as ciliated cells, both *sc-epen-M1 and sn-glia-M4* were associated with GO terms related to 'cilium' in ependymal cells. Module *sc-epen-M2* was characterized by expression of *Vim* (encoding vimentin) which is a shared marker between ependymal cells and tanycytes, while *sc-epen-M9* was comprised of primarily ribosomal genes involved in protein synthesis.

Statistical analysis accompanying main text Figure 6

The conclusion that the effect of icv FGF1 injection to induce sustained remission of hyperglycemia is prevented by continuous icv infusion of the Mc4r antagonist SHU9119 in *ob/ob* mice is supported by

the following statistical analysis. As expected, group means for the glucose area under the curve (AUC glucose) over the entire study period differed significantly across the 4 groups by ANOVA ($F(4,26)=7.2$; $p=5x10^{-4}$), with glucose AUCs lower in the FGF1/SHU9119 group (by 3323 ± 1348, $t(26)=-2.5$; $p=0.021$), and lower still in the FGF1/vehicle group (by 4978 ± 1309, t(26=-3.8; $p=8x10^{-4}$*) vs.* vehicle/vehicle controls (AUC blood glucose = 13108±953 (mg/dl)*d). We also found that the slope of blood glucose over time differed between the two groups receiving icv FGF1 using linear mixed model analysis. Using a likelihood ratio test, the group × time interaction was significant when added to a longitudinal model that included group, time and baseline blood glucose (Chi square (3) = 10.3, $p=0.016$), and the FGF1/SHU9119 group was the only group with an interaction term (e.g., slope of glucose over time) that differed significantly from control (by 5.15 ±1.490 (mg/dl)/day; $z=3.46$; $p=6x10^{-4}$), indicative of a substantial and significant positive slope for blood glucose over the course of the study. Combined with the fact that the FGF1/SHU interaction term was significantly greater than the estimate for the FGF1/vehicle group (by 2.9 ± 1.45 mg/dl/day, $z=1.99$; $p=0.047$), we conclude that after its initial drop, blood glucose levels in the FGF1/SHU9119 group returned toward baseline in a manner that was not observed in the FGF1/vehicle group. Combined with evidence that at the final timepoint, only the FGF1/vehicle group's blood glucose remained significantly below vehicle/vehicle controls (by 109.57 +/- 51.550 mg/dl, $p= 0.033$), we conclude that the effect of a single icv injection of FGF1 to induce sustained diabetes remission was effectively prevented by co-treatment with SHU 9119. We further report that this outcome cannot be attributed to group differences in food intake, since ANOVA indicated that the baseline-adjusted group means for AUC food intake did not differ significantly between the two FGF1-treated groups ($F(4,26)=1.12$; $p = 0.37$), and mixed model analysis

revealed that there were no significant group differences in food intake once the initial, transient anorexic effect of FGF1 had resolved ($p \geq 0.21$).

Supplementary References

1. Lein, E. S. *et al.* Genome-wide atlas of gene expression in the adult mouse brain. *Nature* **445**, 168–176 (2007).
2. Campbell, J. N. *et al.* A molecular census of arcuate hypothalamus and median eminence cell types. *Nat. Neurosci.* **20**, 484-496 (2017).
3. Romanov, R. A. *et al.* Molecular interrogation of hypothalamic organization reveals distinct dopamine neuronal subtypes. *Nat. Neurosci.* **20**, 179-188 (2017).
4. Chen, R., Wu, X., Jiang, L. & Zhang, Y. Single-Cell RNA-Seq Reveals Hypothalamic Cell Diversity. *Cell Rep.* **18**, 3227-3241 (2017). https://doi.org/10.1016/j.celrep.2017.03.004
5. Rinkenberger, J. & Werb, Z. The labyrinthine placenta. *Nature Genetics* **25**, 248–250 (2000).
6. Lee, D. A. *et al.* Tanycytes of the hypothalamic median eminence form a diet-responsive neurogenic niche. *Nat. Neurosci.* **15**, 700–702 (2012).
7. Mizrak, D. *et al.* Single-Cell Analysis of Regional Differences in Adult V-SVZ Neural Stem Cell Lineages. *Cell Rep.* **26**, 394-406.e5 (2019). https://doi.org/10.1016/j.celrep.2018.12.044
8. Diano, S. *et al.* Peroxisome proliferation-associated control of reactive oxygen species sets melanocortin tone and feeding in diet-induced obesity. *Nat. Med.* **17**, 1121–1128 (2011).
9. Elizono-Vega, R. J., Recabal, A. & Oyarce, K. Nutrient sensing by hypothalamic tanycytes. *Front. in Endocrinol.* **10**, 244 (2019). https://doi.org/10.3389/fendo.2019.00244

10. García-Cáceres, C. *et al.* Role of astrocytes, microglia, and tanycytes in brain control of systemic metabolism. *Nat. Neurosci.* **22**, 7–14 (2019).
