# Supplementary material for: Transcriptomic analysis links diverse hypothalamic cell types to fibroblast growth factor 1-induced sustained diabetes remission
Source: Nat Commun. 2020 Sep 7;11:4458. doi: 10.1038/s41467-020-17720-5 (PMC7477234; doi:10.1038/s41467-020-17720-5)
Supplement: Supplementary file 2 — Description of Additional Supplementary Files [file 41467_2020_17720_MOESM2_ESM.docx]

**Description of Additional Supplementary Files**

**Title: Supplementary Data 1**

Description: Differentially expressed genes (DEGs) between pair-fed vehicle and FGF1 treated identified in glia cells by pseudobulk approach in single nuclei RNA-seq data from Day 1. Statistical test is based on Wald test used in the DESeq2 package (Bonferroni adjusted).

Sheets: **a.** astrocytes, **b**. oligodendrocyte precursor cells and committed to differentiate oligodendrocytes, **c**. endothelial cells, **d.** ependymal cells, **e.** microglia, **f.** oligodendrocytes, **g.** tanycytes, **h.** vascular-leptomeningeal cells

**Title: Supplementary Data 2**

Description: Gene ontology (GO) (biological processes; bp), REACTOME (REAC) and Kyoto Encyclopedia of Genes and Genomes (KEGG) results of DEGs (pseudobulk approach) of astrocytes, tanycytes and ependymal cells in the single nuclei RNA-seq data from Day 1.

Sheets: **a.** pathway analysis of differentially expressed genes with |log_2_ fold-change|>1 and FDR<0.05, **b.** pathway analysis of differentially expressed genes with |log_2_ fold-change|<-1 and FDR<0.05

**Title: Supplementary Data 3**

Description: Weighted correlation network analysis (WGCNA) of astrocytes, tanycytes and ependymal cells in the single nuclei RNA-seq data from Days 1 and 5, linear mixed-effects model testing to identify differences between pair-fed (PF) vehicle and FGF1 treated and pathway analysis (GO:bp; REAC; KEGG) of selected modules.

Sheets: **a**. All identified gene modules, **b.** linear mixed-effects model testing, **c.** pathway analysis

**Title: Supplementary Data 4**

Description: WGCNA of ependymal cells in the single cell RNA-seq data from Day 5, linear mixed-effects model testing to identify differences between PF vehicle and FGF1 treated and pathway analysis (GO:bp; REAC; KEGG) of selected modules.

Sheets: **a**. All identified gene modules, **b.** linear mixed-effects model testing, **c.** pathway analysis

**Title: Supplementary Data 5**

Description: WGCNA of tanycytes in the single cell RNA-seq data from Day 5, linear mixed-effects model testing to identify differences between PF vehicle and FGF1 treated and pathway analysis (GO:bp; REAC; KEGG) of selected modules.

Sheets: **a**. All identified gene modules, **b.** linear mixed-effects model testing, **c.** pathway analysis

**Title: Supplementary Data 6**

Description: WGCNA of astrocytes in the single nuclei RNA-seq data from Days 1 and 5, linear mixed-effects model testing to identify differences between PF vehicle and FGF1 treated and pathway analysis (GO:bp; REAC, KEGG) of selected modules.

Sheets: **a**. All identified gene modules, **b.** linear mixed-effects model testing, **c.** pathway analysis

**Title: Supplementary Data 7**

Description: Differentially expressed genes (DEGs) between pair-fed vehicle and FGF1 treated identified in glia cells by pseudobulk approach in single cell RNA-seq data from Day 5. Statistical test is based on Wald test used in the DESeq2 package (Bonferroni adjusted).

Sheets: **a.** astrocytes, **b**. arachnoid barrier cells, **c.** committed to differentiate oligodendrocytes, **d**. endothelial cells, **e.** ependymal cells, **f.** macrophages, **g.** microglia, **h.** oligodendrocytes, **i.** oligodendrocyte precursor cells, **j.** pericyte, **k.** smooth muscle cells, **l.** tanycytes, **m.** vascular-leptomeningeal cells

**Title: Supplementary Data 8**

Description: WGCNA of astrocytes in the single cell RNA-seq data from Day 5, linear mixed-effects model testing to identify differences between PF vehicle and FGF1 treated and pathway analysis (GO:bp; REAC; KEGG) of selected modules.

Sheets: **a**. All identified gene modules, **b.** linear mixed-effects model testing, **c.** pathway analysis

**Title: Supplementary Data 9**

Description: Differentially expressed genes (DEGs) between pair-fed vehicle and FGF1 treated identified in Agrp neurons by pseudobulk approach in single nuclei RNA-seq data from Day 1. Statistical test is based on Wald test used in the DESeq2 package (Bonferroni adjusted).

**Title: Supplementary Data 10**

Description: List of principal component 4 and 5 genes from principal component analysis of Agrp neurons from single nucleus RNA-seq from Days 1 and 5 between pair-fed vehicle and FGF1 in single nuclei RNA-seq data

**Title: Supplementary Data 11**

Description: DEGs between pair-fed vehicle and FGF1 treated identified in bulk RNA-seq data from Days 1, 5 and 42. Statistical test is based on Wald test used in the DESeq2 package (Bonferroni adjusted).

Sheets: **a.** Day 1 DEGs, **b**. Day 5 DEGs, **c**. Day 42 DEGs

**Title: Supplementary Data 12**

Description: Threshold-free geneset enrichment analysis (GO:BP) of DEGs between pair-fed vehicle and FGF1 treated identified in bulk RNA-seq data from Days 1, 5 and 42. Statistics is based on a Kolmogorov-Smirnov-like test used in the GSEA method.
