# Supplementary material for: Transcriptomic analysis links diverse hypothalamic cell types to fibroblast growth factor 1-induced sustained diabetes remission
Source: Nat Commun. 2020 Sep 7;11:4458. doi: 10.1038/s41467-020-17720-5 (PMC7477234; doi:10.1038/s41467-020-17720-5)
Supplement: Supplementary file 15 — Reporting Summary [file 41467_2020_17720_MOESM15_ESM.pdf]

nature research

Corresponding author(s): Tune H. Pers, Michael W. Schwartz

Last updated by author(s): Jun 30, 2020

Reporting Summary

Nature Research wishes to improve the reproducibility of the work that we publish. This form provides structure for consistency and transparency in reporting. For further information on Nature Research policies, see Authors & Referees and the Editorial Policy Checklist .

Statistics

For all statistical analyses, confirm that the following items are present in the figure legend, table legend, main text, or Methods section.

n/a	Confirmed	
☐	☒	The exact sample size (*n*) for each experimental group/condition, given as a discrete number and unit of measurement
☐	☒	A statement on whether measurements were taken from distinct samples or whether the same sample was measured repeatedly
☐	☒	The statistical test(s) used AND whether they are one- or two-sided<br>*Only common tests should be described solely by name; describe more complex techniques in the Methods section.*
☐	☒	A description of all covariates tested
☐	☒	A description of any assumptions or corrections, such as tests of normality and adjustment for multiple comparisons
☐	☒	A full description of the statistical parameters including central tendency (e.g. means) or other basic estimates (e.g. regression coefficient) AND variation (e.g. standard deviation) or associated estimates of uncertainty (e.g. confidence intervals)
☐	☒	For null hypothesis testing, the test statistic (e.g. *F*, *t*, *r*) with confidence intervals, effect sizes, degrees of freedom and *P* value noted<br>*Give P values as exact values whenever suitable.*
☒	☐	For Bayesian analysis, information on the choice of priors and Markov chain Monte Carlo settings
☐	☒	For hierarchical and complex designs, identification of the appropriate level for tests and full reporting of outcomes
☐	☒	Estimates of effect sizes (e.g. Cohen's *d*, Pearson's *r*), indicating how they were calculated

Our web collection on statistics for biologists contains articles on many of the points above.

Software and code

Policy information about availability of computer code

Data collection	No software was used for data collection.
Data analysis	All codes for RNA-seq data analysis are made available through https://perslab.github.io/bentsen-rausch-2020/. The following software was used in this study: Prism ver. 7 (Graph Pad), R ver. 3.5.3 (R Core Team, 2018. https://www.R-project.org/), RStudio ver. 1.1.38, Cell Ranger ver. 1.2 (10x Genomics), Scrublet ver. 0.1, Seurat ver. 2.3 and ver. 3.0, DESeq2 ver. 1.22.2, gProfiler ver. 0.6.7, WGCNA ver. 1.66, nlme ver. 3.1-140, princurve ver. 2.1.4, STAR aligner ver. 2.5.4b, Fiji ver. 1.51j8 (NIH), Imaris ver. 9.1.0 (Bitplane), HALO ISH-IHC ver. 1.3 (Indica Labs).

For manuscripts utilizing custom algorithms or software that are central to the research but not yet described in published literature, software must be made available to editors/reviewers. We strongly encourage code deposition in a community repository (e.g. GitHub). See the Nature Research guidelines for submitting code & software for further information.

Data

Policy information about availability of data

All manuscripts must include a data availability statement. This statement should provide the following information, where applicable:

- Accession codes, unique identifiers, or web links for publicly available datasets
- A list of figures that have associated raw data
- A description of any restrictions on data availability

All RNA-seq data generated in this study (snRNA-seq, scRNA-seq and bulk RNA-seq) are available through the NCBI Gene Expression Omnibus (GEO) under Super Series accession number GSE153551 (https://www.ncbi.nlm.nih.gov/geo/query/acc.cgi?acc=GSE153551). This study also used publicly available datasets including ‘A Molecular Census of Arcuate Hypothalamus and Median Eminence Cell Types’ (GSE93374; https://www.ncbi.nlm.nih.gov/geo/query/acc.cgi?acc=GSE93374), ‘Single-cell RNA-seq of mouse hypothalamus’ (GSE74672; https://www.ncbi.nlm.nih.gov/geo/query/acc.cgi?acc=GSE74672), ‘Single-cell RNA-seq reveals hypothalamic cell diversity’ (GSE87544; https://www.ncbi.nlm.nih.gov/geo/query/acc.cgi?acc=GSE87544), ‘Expression data from reactive astrocytes purified from young adult mouse brains’ (GSE35338; https://www.ncbi.nlm.nih.gov/geo/query/acc.cgi?acc=GSE35338), ‘RNA-seq analysis of single cells of the oligodendrocyte

lineage from nine distinct regions of the anterior-posterior and dorsal-ventral axis of the mouse juvenile central nervous system' (GSE75330; https://www.ncbi.nlm.nih.gov/geo/query/acc.cgi?acc=GSE75330), and 'RNA-seq of mouse astrocytes in monoculture and co-cultured with rat neurons (both inactive and active)' (E-MTAB-5514; https://www.ebi.ac.uk/arrayexpress/experiments/E-MTAB-5514/). The authors declare that all the data supporting the findings of this study are available within the article and its supplementary information files or from the corresponding authors upon request. Source data are provided with this paper including data underlying Figs. 1a, 2e, 3g, 4b, 4f, 5h, 5i, 6a, 6b, 6c, 7e, 7g and Supplementary Fig. 6. Images are available upon request from the corresponding authors.

Field-specific reporting

Please select the one below that is the best fit for your research. If you are not sure, read the appropriate sections before making your selection.

[x] Life sciences [ ] Behavioural & social sciences [ ] Ecological, evolutionary & environmental sciences

For a reference copy of the document with all sections, see nature.com/documents/nr-reporting-summary-flat.pdf

Life sciences study design

All studies must disclose on these points even when the disclosure is negative.

Sample size	Sample size for each study was determined based on published (Scarlett et al., 2016; Campbell et al., 2017) and prior work by the authors.
Data exclusions	None
Replication	Seven independent cohorts of mice were used to confirm findings across methodologies and cohorts, and all seven replications were successful.
Randomization	Allocation of mice to groups were based on morning blood glucose measures and body weight to match the mean of both groups
Blinding	Samples were de-identified with the investigator performing downstream processing and blinded to the study groups

Reporting for specific materials, systems and methods

We require information from authors about some types of materials, experimental systems and methods used in many studies. Here, indicate whether each material, system or method listed is relevant to your study. If you are not sure if a list item applies to your research, read the appropriate section before selecting a response.

Materials & experimental systems

n/a	Involved in the study	
[ ]	[x]	Antibodies
[x]	[ ]	Eukaryotic cell lines
[x]	[ ]	Palaeontology
[ ]	[x]	Animals and other organisms
[x]	[ ]	Human research participants
[x]	[ ]	Clinical data

Methods

n/a	Involved in the study	
[x]	[ ]	ChIP-seq
[x]	[ ]	Flow cytometry
[x]	[ ]	MRI-based neuroimaging

Antibodies

Antibodies used	chicken anti-vimentin (1:500, EMD Millipore, AB5733), mouse anti-GFAP (1:500, EMD Millipore, MAB3402), rabbit anti-Aquaporin 4 (1:200, EMD Millipore, AB3594), rabbit anti NPY (1:1,500, Abcam, ab30914), rabbit anti-AGRP (1:1000, Phoenix Pharmaceuticals, Inc, 003-53), Alexa dyes (goat or donkey polyclonal, 1:500, Invitrogen), donkey anti-rabbit Alexa 594 (1:500; Invitrogen, A-21207), goat anti-chicken Alexa 555 (1:500; Invitrogen, A11039), goat anti-mouse Alexa 488 (1:500; Invitrogen, A28175)
Validation	chicken anti-vimentin: 36 citations on labome.com including 8 with use in IHC in mouse (https://www.labome.com/product/EMD-Millipore/AB5733.html); mouse anti-GFAP: 7 confirmed immunohistochemistry citations on manufacturer's webpage (merckmillipore.com/DK/en/product/Anti-Glial-Fibrillary-Acidic-Protein-Antibody-clone-GA5,MM_NF-MAB3402#documentation) and 20 published figures on Benchsci (https://app.benchsci.com/product/EMD%20Millipore/MAB3402/figures); rabbit anti-Aquaporin 4: 3 citations on manufacturer's website (http://www.emdmillipore.com/US/en/product/Anti-Aquaporin-4-Antibody-CT,MM_NF-AB3594-50UL)); rabbit anti-AGRP: 8 citations on manufacturer's webpage (https://www.phoenixpeptide.com/products/view/Antibodies/H-003-53); rabbit anti-NPY: 4 citations on citeab (https://www.citeab.com/antibodies/779139-ab30914-anti-neuropeptide-y-antibody)

Animals and other organisms

Policy information about studies involving animals; ARRIVE guidelines recommended for reporting animal research

Laboratory animals	Mice were individually housed under specific-pathogen free conditions in a temperature-controlled room, with 75-80% humidity and with a 12:12h light:dark cycle. Mice were provided with ad libitum access to water and Purina 5008 chow (Animal Specialties, Inc., CA, USA) or Altromin 1310 chow (Brogaarden, Denmark) unless otherwise stated. Lep(ob/ob) (male, 6-8 weeks) were used to perform bulk RNA-seq, single nuclei RNA-seq analysis, immunohistochemistry (vimentin, Gfap, Aqp4), electron microscopy and for evaluation of the effect of SHU9119 (Jackson Labs, USA) and single cell RNA-seq (Janivier Labs, France). KK-Ay mice (male, 6-8 weeks; Jackson Labs, USA) were used to study the effect of FGF1 in hyperglycemic Agouti mice. Mc4r-/- (male, 6-8 weeks; Jackson Labs, USA) were used to study if the effect of FGF1 to lower blood glucose in a sustained manner is melanocortin signaling dependent. Mice were allowed to acclimatize at leaset 7 days before surgery, and allowed to recover for 2 weeks or more after surgery.
Wild animals	The study did not involve use of wild animals.
Field-collected samples	The study did not involve use of field-collected samples.
Ethics oversight	All procedures were either performed in accordance with the NIH Guide for the Care and Use of Laboratory Animals and were approved by the Institutional Animal Care and Use Committee at the University of Washington, Novo Nordisk Research Center Seattle and St. Joseph's Hospital and Medical Center, or performed with approved protocols from the The Danish Animal Experiments Inspectorate permit number 2014-15-0201-00181 and the University of Copenhagen project number P16-122.

Note that full information on the approval of the study protocol must also be provided in the manuscript.
